# Glycopolymers Prepared by Alternating Ring-Opening Metathesis Polymerization Provide Access to Distinct, Multivalent Structures for the Probing of Biological Activity

**DOI:** 10.1021/acsbiomedchemau.4c00018

**Published:** 2024-05-28

**Authors:** Luz C. Mendez, Francis O. Boadi, Mitchell Kennedy, Surita R. Bhatia, Nicole S. Sampson

**Affiliations:** † Department of Chemistry, Stony Brook University, Stony Brook, New York 11794-3400, United States; ‡ Department of Chemistry, University of Rochester, Rochester, New York 14627-0216, United States

**Keywords:** ruthenium catalysis, small-angle X-ray scattering, polyvalent, functional polymer, copolymer

## Abstract

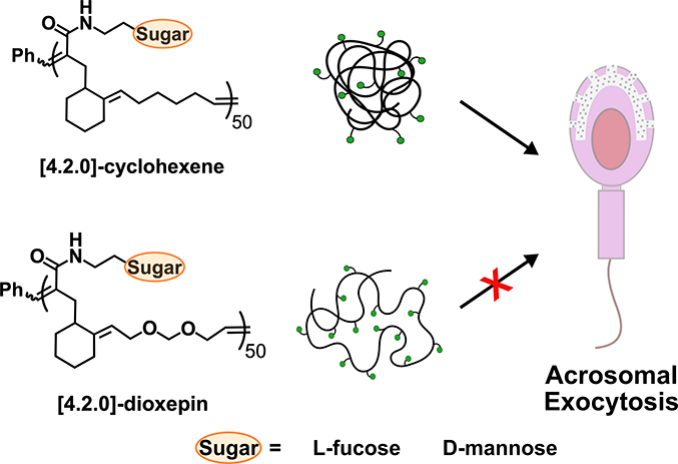

A myriad of biological processes are facilitated by ligand–receptor interactions. The low affinities of these interactions are typically enhanced by multivalent engagements to promote binding. However, each biological interaction requires a unique display and orientation of ligands. Therefore, the availability and diversity of synthetic multivalent probes are invaluable to the investigation of ligand–receptor binding interactions. Here, we report glycopolymers prepared from bicyclo[4.2.0]oct-6-ene-7-carboxamide and 4,7-dihydro-1,3-dioxepin or cyclohexene. These glycopolymers, synthesized by alternating ring-opening metathesis polymerization, display precise ligand spacing as well as the option of a hydrophobic or acetal-functionalized polymer backbone. Small-angle X-ray scattering (SAXS) data analysis revealed that these [4.2.0] glycopolymers adopted distinct conformations in solution. In aqueous media, [4.2.0]-dioxepin glycopolymers formed swollen polymer chains with rod-like, flexible structures while [4.2.0]-cyclohexene glycopolymers assumed compact, globular structures. To illustrate how these glycopolymers could aid in the exploration of ligand–receptor interactions, we incorporated the [4.2.0] glycopolymers into a biological assay to assess their potential as activators of acrosomal exocytosis (AE) in mouse sperm. The results of the biological assay confirmed that the differing structures of the [4.2.0] glycopolymers would evoke distinct biological responses; [4.2.0]-cyclohexene glycopolymers induced AE in mouse sperm while [4.2.0]-dioxepin glycopolymers did not. Herein, we provide two options for glycopolymers with low to moderate molecular weight dispersities and low cytotoxicity that can be implemented into biological assays based on the desired hydrophobicity, rigidity, and structural conformation of the polymer probe.

Recent developments in the methodology of polymerizations have broadened the scope of synthetic macromolecules available. In particular, major strides have been taken to advance the syntheses of polymers and simultaneously improve the breadth of functionality and applications of these structures in biological systems. Evolution in free radical polymerization^1^ and atom transfer radical polymerization^2^ has resulted in biocompatible polymers with increased water solubility and low cytotoxicity. The utilization of polycondensation or ring-opening polymerization to synthesize polyesters has inspired the development of biocompatible materials for dental, orthopedic, drug delivery, and heart regeneration applications.^3−6^ In our laboratory, we have applied alternating ring-opening metathesis polymerization (AROMP) methods to synthesize sequence-controlled polymers with tunable hydrophilicity, morphology, and degradability.^7−9^

Our previous work on alternating copolymers prepared from bicyclo[4.2.0]oct-1(8)-ene-8-carboxamide and cyclohexene via AROMP-provided structures with low-molecular-weight dispersities and a high degree of polymerization (DP_*n*_).^10^ Likewise, our work demonstrating the AROMP of cyclic acetal or lactone monomers with bicyclo[4.2.0]oct-1(8)-ene-8-carboxamide resulted in the synthesis of polymers with low to moderate molecular weight distributions and acetal or ester-functionalized backbones.^9^ The outcomes of these studies encouraged our group to modify these polymers with additional functionality and potential for biological applications. In particular, we sought to produce polymers that could be implemented in the investigation of biological processes that are dependent on receptor–ligand interactions and multivalency. Multivalent engagements are ubiquitous in fertilization, virus-cell binding, antibody–antigen binding, and cell–cell signaling.^11−14^ However, each biological interaction requires a specific display of multivalent ligands to achieve efficient binding typically not observed in low-affinity monovalent interactions.^12,14^ Synthetic multivalent ligands, or polymer probes, provide an array of tunable ligand displays that have been shown to act as excellent effectors or inhibitors of biological activity through receptor clustering, chelation, and increased local concentration near target receptors.^15−18^ Additionally, multivalent displays have been shown to improve binding avidity and specificity when properties such as polymer flexibility, conformation, valency, or molecular weight are manipulated.^19−22^

In this work, bicyclo[4.2.0]oct-6-ene-7-carboxamide bearing mannose or fucose was allowed to react with cyclohexene or 4,7-dihydro-1,3-dioxepin via ruthenium-catalyzed AROMP. These [4.2.0] glycocopolymers were synthesized to produce polymer probes with unique ligand spacing and options for backbone hydrophilicity and compared to their norbornyl counterparts (Figure 1). Small-angle X-ray scattering (SAXS) was employed to ascertain the conformations of these glycopolymers in solution and identify structural differences arising from the polymer backbones. Finally, we demonstrated that these [4.2.0] glycopolymers could be incorporated into a biological assay to probe the activation of signaling pathways. We compared [4.2.0] glycopolymers displaying fucose or mannose sugars to our previous polynorbornene glycopolymers^23^ and determined their effects in activating acrosomal exocytosis (AE), an essential phenomenon in mammalian fertilization. By diversifying our library of glycopolymers with the [4.2.0] structures, we were able to test polymers with a hydrophilic backbone, which was not accessible in our previous experiments.^23−25^ Herein, we report novel [4.2.0] glycopolymers with low to moderate molecular weight dispersities and low cytotoxicity that can be implemented into biological assays dependent on the structural conformations required to probe a receptor–ligand interaction of interest.

**Figure 1:**
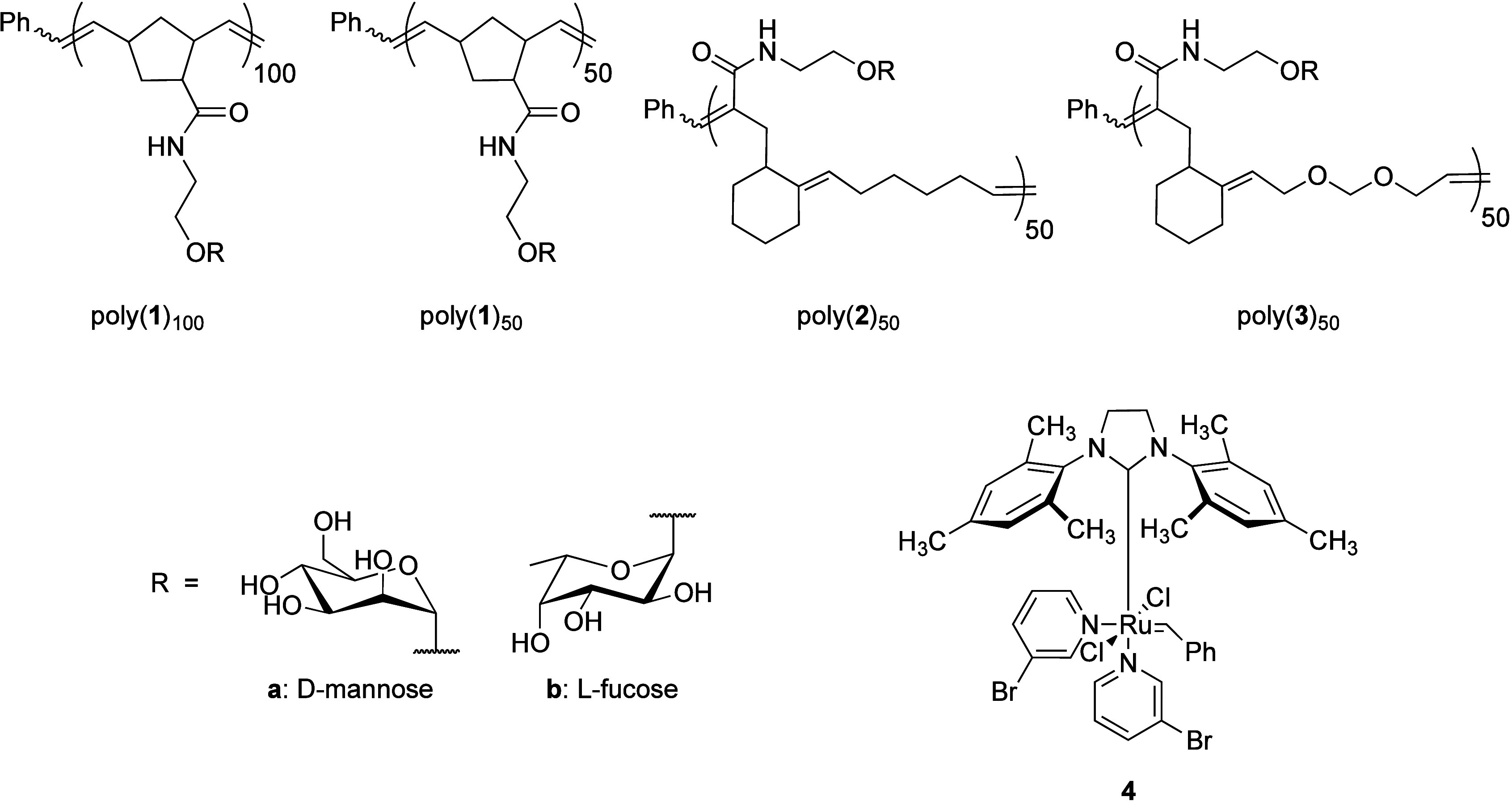
Backbone structures of polynorbornene and [4.2.0] glycocopolymers. Poly(**1**)_100_ and poly(**1**)_50_ represent polynorbornene structures. Poly(**2**)_50_ and poly(**3**)_50_ correspond to [4.2.0]-cyclohexene and [4.2.0]-dioxepin glycopolymers, respectively. Grubbs’ third-generation catalyst **4** was used in the polymerization of norbornene and [4.2.0] sugar monomers.

## Materials and Methods

### Materials

All experiments performed on mice were approved by the Stony Brook University IACUC (Protocol 252156) and were conducted in accordance with the National Institute of Health and the United States Department of Agriculture guidelines. Anhydrous dimethyl sulfoxide (DMSO), soybean trypsin inhibitor (SBTI), propidium iodide (PI), and bovine serum albumin (BSA), fraction V were purchased from Sigma-Aldrich (St. Louis, MO). Alexa Fluor 488 tetrafluorophenyl (TFP) ester, SYTO 17, and Dulbecco’s phosphate-buffered saline (DPBS) were purchased from Life Technologies (Carlsbad, CA). All other chemicals and supplies were purchased from Sigma-Aldrich, Fisher Scientific (Hampton, NH), or VWR (Radnor, PA).

### Fluorescent Stain Preparation and Storage

PI was purchased as a stock solution dissolved in water (2.4 mM) and stored at 4 °C. A 5 mM SYTO 17 solution was diluted in anhydrous DMSO (1 mM) and stored at −20 °C as aliquots. Alexa Fluor 488 soybean trypsin inhibitor conjugates (SBTI-Alexa 488) were prepared in 1× DPBS buffer containing 0.02% sodium azide (Table S1) and stored at −20 °C as aliquots. The concentration and degree of labeling of protein conjugates was determined by UV–vis spectroscopy (Figure S1).

### Glycopolymer Solution Storage

Norbornene glycopolymers were synthesized, purified, and characterized as previously reported.^23^ Stock solutions of poly(**1**)_100_, poly(**1**)_50_, poly(**2**)_50_, and poly(**3**)_50_ were dissolved in distilled deionized (ddI) water and stored at −20 °C at a polymer concentration of 100 μM.

### Components of M16 Buffer

Phenol red-free M16 buffer was prepared by combining the following reagents in 20 mL of ddI water: 94.6 mM NaCl, 4.8 mM KCl, 1.2 mM KH_2_PO_4_, 1.2 mM MgSO_4_·7H_2_O, 38.8 mM of sodium lactate (syrup, 60% w/w), 5.6 mM d-glucose, 0.0006% penicillin K^+^ salt, 0.0005% streptomycin sulfate, 25.0 mM NaHCO_3_, 0.33 mM sodium pyruvate, and 2.0 mM CaCl_2_·2H_2_O. The buffer was then supplemented with 0.3% (w/v) BSA, fraction V and filtered through a 0.22 μm poly(ether sulfone) (PES) sterile membrane. A fresh solution of M16 medium was prepared before every experiment.

### Sperm Acquisition and Capacitation

Mouse sperm acquisition, treatment, and flow cytometry experiments were adapted from previously published methods.^24^ Sperm were forced out of the cauda epididymis of two 9-to-12-week-old CD-1 male mice (Charles River) and into M16 medium (6 mL) supplemented with 0.3% (w/v) BSA, fraction V. Sperm were allowed to swim out of the epididymis for 10 min at 25 °C. The sperm suspension was then gently pipetted into a polypropylene conical tube (30 × 115 mm) and allowed to incubate at 25 °C for 10 min. Sperm concentration was assessed by a hemocytometer, and sperm motility was observed by phase-contrast microscopy (20×). After incubation, aliquots of sperm (125 μL) containing approximately 2.5 × 10^6^ cells were transferred to polypropylene culture tubes (12 × 75 mm) and diluted to 250 μL with M16 medium. The sperm were allowed to incubate for an additional 15 min at 25 °C to promote capacitation.

### Sperm Treatment

A 250 μL solution containing SYTO 17 (5 μM) and SBTI-Alexa 488 (2 μg/μL) was prepared using DPBS. A negative control or glycopolymer sample was prepared by adding DPBS or 0.1–20 μM glycopolymer, respectively. The 250 μL solution was then added to the 250 μL sperm suspension for a final volume of 500 μL. The sample was then incubated for 25 min at 25 °C in the dark. After incubation, samples were centrifuged for 5 min at 500 g and the supernatant was aspirated. The resulting pellet was then resuspended with 500 μL of DPBS containing PI (24 μM), SYTO 17 (5 μM), and SBTI-Alexa 488 (2 μg/μL). Pellets from each sample were resuspended at 1:30 min intervals and allowed to incubate for 20 min at 25 °C in the dark before analysis by flow cytometry.

### Assessment of Acrosomal Exocytosis by Flow Cytometry

Flow cytometry and data analysis were performed on a BD LSRFortessa Cell Analyzer in combination with the BD FACSDiva Software Version 9.0 (BD Biosciences). SYTO 17 was used to stain all cellular events and was excited using a 640 nm laser and detected at 670/30 nm. PI stained all nonviable cells and was excited using a 561 nm laser and detected at 582/15 nm. SBTI-Alexa 488 was used to label cells that had undergone acrosomal exocytosis. SBTI-Alexa 488 was excited using the 488 nm laser and detected at 530/30 nm. Gating of noncellular events and weak fluorescent staining was performed as previously published.^24^ Sperm viability and acrosome integrity were measured at a flow rate of 600–800 cells/sec for 1:30 min. 50,000 events were recorded for each sample. Only live sperm (PI negative) were used in further analyses.

### Statistical Analysis

In the presence of DPBS (negative control), 7.2% of sperm underwent AE. When treated with efficient glycopolymers, the maximum AE activity expected was about 17%.^23−25^ The AE% induced by each glycopolymer at every concentration was compared to the AE% induced by DPBS. The AE% of two consecutive concentrations of polymer was also compared. Significant differences in AE% were calculated using one-way analysis of variance (ANOVA) with GraphPad Prism 10. The data represents the mean ± standard error of the mean of at least three independent experiments using two separate batches of polymer. For any statistical significance, *p < 0.05, **p < 0.01, ***p < 0.001, and ****p < 0.0001.

### Gel Permeation Chromatography (GPC)

Analysis was performed on a system comprising a Shimadzu SCL-10A controller, a Shimadzu LC–20AT pump, and a Shimadzu CTO-10AS column oven equipped with two combined Phenogel columns: 5 μm 50 Å (300 × 4.6 mm, 100–3k) and 5 μm 10E3 Å (300 × 4.6 mm, 1k–75k). This was coupled with a Brookhaven Instruments BI-DNDC refractometer. The mobile phase used was HPLC-grade tetrahydrofuran (THF) filtered through a 0.2 μm nylon membrane. The protected polymers were dissolved in the same mobile phase and filtered through a 0.45 μm polytetrafluoroethylene (PTFE) membrane before 100 μL of the sample was injected into the system. Analysis was then performed at 30 °C and with a flow rate of 0.35 mL min^–1^. Polystyrene was used as a standard for calibration.

### Preparation of 2,3,4,6-Tetra-*O*-acetyl-α-d-mannopyranosyl Bicyclo[4.2.0]oct-6-ene-7-carboxamide

1,2,3,4,6-Penta-*O*-acetyl-α-D-mannopyranose (500 mg, 1.28 mmol, 1 equiv) and *N*-(2-hydroxyethyl)bicyclo[4.2.0]oct-6-ene-7-carboxamide (500 mg, 2.56 mmol, 2 equiv) were added to a 25 mL round-bottom flask and subjected to a vacuum-nitrogen cycle (3×). The contents of the flask were then dissolved in 8 mL of acetonitrile and flushed with additional nitrogen gas for 5 min. The flask was chilled at 0 °C, and boron trifluoride etherate (557 μL, 4.48 mmol, 3.5 equiv) was added dropwise. The reaction mixture was left to stir for 18 h. The reaction was then quenched with triethylamine (1.0 mL), concentrated, and redissolved in 100 mL of dichloromethane. The crude mixture was washed with saturated NaHCO_3_ (30 mL), DI H_2_O (30 mL), and brine solution (30 mL). The organic layer was dried over anhydrous MgSO_4_ and concentrated in vacuo. The mixture was then purified by flash chromatography on silica using 4:1 (v/v) ethyl acetate:hexanes to afford the desired product (yield: 30%, 202 mg). ^1^H NMR (700 MHz, CDCl_3_) δ 5.85 (q, 1H, *J* = 6.5 Hz), 5.35 (dd, 1H, *J* = 10.0, 3.5 Hz), 5.33 (m, 2H), 4.85 (d, 1H, *J* = 2.1 Hz), 4.28 (td, 1H, *J* = 11.9, 5.8 Hz), 4.13 (ddd, 1H, *J* = 12.2, 4.5, 2.1 Hz), 3.98 (ddt, 1H, *J* = 9.8, 5.9, 2.6 Hz), 3.83 (m, 1H), 3.57 (m, 2H), 2.88 (m, 1H), 2.73 (tt, 1H, *J* = 15.7, 4.0 Hz), 2.39 (dt, 1H, *J* = 11.1, 4.6 Hz), 2.23 (m, 1H), 2.18 (s, 3H), 2.12 (d, 4H, *J* = 2.0 Hz), 2.07 (s, 3H), 2.03 (s, 3H), 1.95 (m, 1H), 1.77 (m, 1H), 1.35 (m, 2H), 1.15 (m, 1H). ^13^C NMR (176 MHz, CDCl_3_) δ 170.67, 170.02, 169.66, 164.14, 97.62, 69.38, 69.03, 69.01, 68.81, 68.77, 67.49, 67.37, 66.05, 62.39, 38.40, 37.91, 33.93, 33.90, 32.93, 32.87, 27.32, 27.29, 26.73, 26.71, 24.63, 20.90, 20.75, 20.72. HRMS (*m*/*z*) calcd [M + H]^+^: 526.2290, found: 526.2282.

### Preparation of 2,3,4-Tri-*O*-acetyl-l-fucopyranosyl Bicyclo[4.2.0]oct-6-ene-7-carboxamide

1,2,3,4-Tetra-*O*-acetyl-α-L-fucopyranose (240 mg, 0.72 mmol, 1 equiv) and *N*-(2-hydroxyethyl)bicyclo[4.2.0]oct-6-ene-7-carboxamide (280 mg, 1.44 mmol, 2 equiv) were added to a 25 mL round-bottom flask and subjected to a vacuum-nitrogen cycle (3×). The contents of the flask were then dissolved in 4 mL of acetonitrile. After flushing the flask with additional nitrogen for 5 min, the reaction mixture was chilled at 0 °C. Boron trifluoride etherate (311 μL, 2.5 mmol, 3.5 equiv) was then added dropwise. The reaction mixture was left to stir for 18 h, and triethylamine (0.8 mL) was then added to quench the reaction. The mixture was then concentrated and redissolved in 70 mL of dichloromethane. The crude mixture was washed with saturated NaHCO_3_ (15 mL), DI H_2_O (15 mL), and brine solution (15 mL). The organic layer was then dried over anhydrous MgSO_4_ and concentrated in vacuo. The mixture was purified by flash chromatography on silica with 4:1 (v/v) ethyl acetate:hexanes to afford the β-rich product, with C-1 β-anomer ≥90%. Note: To achieve higher purity, the product was purified twice by flash chromatography (yield: 57%, 236 mg). ^1^H NMR (700 MHz, CDCl_3_) δ 5.83 (q, 1H, *J* = 5.8 Hz), 5.26 (d, 1H, *J* = 3.5 Hz), 5.19 (dd, 1H, *J* = 10.5, 8.2 Hz), 5.03 (ddd, 1H, *J* = 10.5, 3.4, 1.5 Hz), 4.47 (d, 1H, *J* = 8.0 Hz), 3.93 (m, 1H), 3.83 (q, 1H, *J* = 6.5 Hz), 3.72 (m, 1H), 3.58 (m, 1H), 3.47 (m, 1H), 2.89 (td, 1H, *J* = 14.5, 2.9 Hz), 2.69 (m, 1H), 2.37 (dt, 1H, *J* = 10.5, 5.0 Hz), 2.22 (m, 1H), 2.20 (s, 3H), 2.16–2.08 (m, 2H), 2.06 (s, 3H), 2.01 (s, 3H), 1.94 (m, 1H), 1.76 (m, 1H), 1.39–1.26 (m, 3H), 1.24 (d, 3H, *J* = 6.3 Hz), 1.13 (m, 1H). ^13^C NMR (176 MHz, CDCl_3_) δ 170.64, 170.20, 169.45, 164.17, 162.10, 126.37, 126.29, 100.88, 72.69, 72.66, 71.90, 71.33, 71.32, 69.23, 69.20, 68.31, 61.94, 61.92, 38.56, 38.52, 37.87, 37.84, 33.87, 32.90, 27.22, 26.71, 24.62, 20.74, 20.70, 20.61. HRMS (*m*/*z*) calcd [M + H]^+^: 468.2235, found: 468.2229.

## General Preparation of Poly(**2′**)_50_

To a septum-sealed vial containing nitrogen and a stir bar, an aliquot (280 μL) of **4** stock solution (0.013 M) in dichloromethane was added (0.0059 M, 1 equiv). A solution of 2,3,4,6-tetra-*O*-acetyl-α-d-mannopyranosyl bicyclo[4.2.0]oct-6-ene-7-carboxamide or 2,3,4,-tri-*O*-acetyl-l-fucopyranosyl bicyclo[4.2.0]oct-6-ene-7-carboxamide (0.29 M, 50 equiv) in dichloromethane (300 μL) was added to the initiator in the vial, and the reaction was stirred at 40 °C for 15 min. Cyclohexene (37 μL, 0.59 M, 100 equiv) was added dropwise to the mixture, and the reaction was stirred at 40 °C for an additional 23 h. The reaction was then terminated by addition of excess ethyl vinyl ether (0.2 mL). The polymer was precipitated with diethyl ether chilled to −20 °C to afford a brown solid and then dried under reduced pressure to remove residual solvents. The acetylated polymer was then analyzed by GPC and NMR to determine dispersity and purity.

Poly(**2a′**)_50_: ^1^H NMR (500 MHz, CD_2_Cl_2_) δ 6.22 (s, 1H), 6.15 (t, 1H, *J* = 4.3 Hz), 5.24 (m, 3H), 5.06 (m, 1H), 4.82 (t, 1H, *J* = 3.1 Hz), 4.22 (ddd, 1H, *J* = 12.1, 5.7, 4.1 Hz), 4.06 (dt, 1H, *J* = 12.3, 2.4 Hz), 3.98 (ddd, 1H, *J* = 9.3, 5.6, 2.6 Hz), 3.78 (dt, 1H, *J* = 9.1, 4.7 Hz), 3.55 (ddd, 2H, *J* = 17.1, 11.9, 4.9 Hz), 2.52 (ddt, 1H, *J* = 21.1, 14.9, 6.3 Hz), 2.36 (dt, 1H, *J* = 13.7, 7.5 Hz), 1.90 (m, 18H), 1.63 (s, 5H), 1.37 (m, 7H). ^13^C NMR (125 MHz, CD_2_Cl_2_) δ 170.83, 170.27, 170.22, 169.99, 136.69, 135.93, 121.08, 98.14, 69.70, 69.56, 69.17, 67.77, 66.41, 62.79, 44.05, 39.61, 30.40, 29.24, 28.76, 28.46, 27.38, 21.01, 20.91, 20.88.

Poly(**2b′**)_50_: ^1^H NMR (700 MHz, CD_2_Cl_2_) δ 6.21 (m, 1H), 6.15 (q, 1H, *J* = 7.5 Hz), 5.24 (dd, 1H, *J* = 3.6, 1.2 Hz), 5.10 (td, 2H, *J* = 10.1, 9.5, 6.9 Hz), 5.02 (dd, 1H, *J* = 10.5, 3.5 Hz), 4.50 (ddd, 1H, *J* = 7.8, 4.6, 2.4 Hz), 3.86 (dt, 2H, *J* = 10.4, 4.5 Hz), 3.72 (dq, 1H, *J* = 10.3, 5.1 Hz), 2.56 (m, 1H), 2.39 (dt, 1H, *J* = 12.8, 9.1 Hz), 2.24 (m, 1H), 2.19–1.96 (m, 15H), 1.83–1.56 (m, 5H), 1.51–1.30 (m, 7H), 1.21 (d, 3H, *J* = 6.4 Hz). ^13^C NMR (176 MHz, CD_2_Cl_2_) δ 170.43, 170.00, 169.80, 169.76, 169.46, 141.87, 136.67, 136.58, 135.02, 134.91, 120.69, 101.12, 101.08, 71.27, 70.13, 69.17, 68.86, 43.67, 43.59, 39.40, 32.96, 30.00, 29.91, 28.87, 28.35, 28.08, 26.97, 26.73, 26.70, 23.87, 20.62, 20.42, 15.90, 15.85.

### General Preparation of Poly(**3′**)_50_

To a septum-sealed vial containing nitrogen and a stir bar, a solution of **4** (0.0072 M, 1 equiv) in dichloromethane (50 μL) was added. A solution of 2,3,4,6-tetra-*O*-acetyl-α-d-mannopyranosyl bicyclo[4.2.0]oct-6-ene-7-carboxamide or 2,3,4,-tri-*O*-acetyl-l-fucopyranosyl bicyclo[4.2.0]oct-6-ene-7-carboxamide (0.36 M, 50 equiv) in dichloromethane (450 μL) was then added to the vial, and the reaction was initiated at 40 °C for 15 min. 4,7-Dihydro-1,3-dioxepin (31 μL, 0.54 M, 75 equiv) diluted in dichloromethane (100 μL total volume) was added dropwise to the mixture, and the reaction was stirred for an additional 20 h at 40 °C. The reaction was terminated by addition of excess ethyl vinyl ether (0.2 mL). The polymer was precipitated with diethyl ether chilled to −20 °C to afford a brown solid and then dried under reduced pressure to remove residual solvents. The acetylated polymer was then analyzed by GPC and NMR to determine dispersity and purity.

Poly(**3a′**)_50_: ^1^H NMR (700 MHz, CD_2_Cl_2_) δ 7.29 (s, 1H), 6.43 (s, 1H), 6.22 (s, 1H), 5.85 (s, 1H), 5.74 (s, 1H), 5.40 (s, 2H), 5.37 (s, 1H), 5.35–5.23 (m, 5H), 5.23 (s, 3H), 5.02 (d, 1H, *J* = 11.2 Hz), 4.89–4.86 (m, 1H), 4.86 (s, 2H), 4.76–4.66 (m, 5H), 4.30–4.21 (m, 3H), 4.16–4.12 (m, 1H), 4.12 (s, 1H), 4.10 (s, 5H), 4.04–3.99 (m, 3H), 3.82 (dq, 2H, *J* = 10.3, 6.1, 5.5 Hz), 3.61 (s, 4H), 3.53–3.44 (m, 1H), 2.66 (s, 1H), 2.38 (dd, 1H, *J* = 14.5, 8.1 Hz), 2.18 (s, 5H), 2.13 (s, 1H), 2.07 (d, 12H, *J* = 25.2 Hz), 1.70 (s, 4H), 1.64 (s, 5H), 1.57 (s, 3H), 1.49 (s, 4H), 1.36 (s, 2H), 1.19 (t, 1H, *J* = 7.0 Hz). ^13^C NMR (176 MHz, CD_2_Cl_2_) δ 170.42, 169.88, 169.81, 169.61, 168.03, 150.85, 138.48, 130.89, 128.95, 116.82, 97.70, 97.59, 96.32, 93.70, 69.32, 69.28, 69.11, 68.75, 68.32, 67.16, 66.01, 63.78, 62.85, 62.39, 43.45, 43.25, 39.25, 39.17, 39.09, 32.80, 32.45, 30.40, 28.08, 27.93, 27.20, 23.71, 20.61, 20.50, 20.48.

Poly(**3b′**)_50_: ^1^H NMR (700 MHz, CD_2_Cl_2_) δ 6.35 (s, 1H), 6.21 (s, 1H), 5.35 (d, 4H, *J* = 1.9 Hz), 5.30 (s, 3H), 5.24 (d, 2H, *J* = 3.8 Hz), 5.11 (d, 2H, *J* = 10.1 Hz), 5.0–4.98 (m, 3H), 4.68 (q, 3H, *J* = 14.2, 9.8 Hz), 4.50 (d, 2H, *J* = 7.8 Hz), 4.21 (s, 2H), 4.14 (s, 2H), 4.08 (d, 2H, *J* = 18.4 Hz), 3.86 (s, 3H), 3.73 (s, 2H), 3.47 (ddd, 2H, *J* = 10.6, 7.4, 4.1 Hz), 2.66 (s, 1H), 2.38 (s, 2H), 2.30 (s, 2H), 2.19 (d, 1H, *J* = 7.5 Hz), 2.18 (s, 3H), 2.12 (s, 2H), 2.05 (d, 2H, *J* = 10.3 Hz), 1.99 (d, 4H, *J* = 3.8 Hz), 1.70 (s, 2H), 1.65–1.62 (m, 16H), 1.50 (s, 3H), 1.35 (s, 2H), 1.20 (dt, 4H, *J* = 28.9, 6.2 Hz). ^13^C NMR (176 MHz, CD_2_Cl_2_) δ 170.47, 170.01, 169.60, 169.55, 169.13, 150.45, 138.81, 130.54, 128.98, 117.18, 101.09, 96.30, 93.71, 71.26, 70.12, 69.22, 69.17, 68.91, 68.85, 68.71, 68.63, 68.33, 67.17, 63.81, 62.87, 43.40, 43.23, 43.18, 39.49, 39.29, 32.94, 32.80, 32.46, 30.37, 28.26, 28.12, 27.95, 27.17, 23.68, 20.60, 20.42, 15.87, 15.83.

### General Preparation of Poly(**2**)_50_ and Poly(**3**)_50_

Poly(**2′**)_50_ or poly(**3′**)_50_ (30 mg) was added to a reaction vial and dissolved in 0.5 mL of THF. One milliliter of a mixture of MeOH:H_2_O (2:1.5 v/v) containing K_2_CO_3_ (in excess) was then added. The reaction was allowed to stir at 25 °C for 3 h. The reaction mixture was neutralized with 0.5 mL of 1 N HCl in H_2_O:THF (1:1 v/v). The solution was then transferred to a prewetted cellulose ester Spectra/Por Float-A-Lyzer G2 dialysis device (MWCO 500–1kD, 5 mL) and dialyzed against DI water for at least 3 d. The mixture was then lyophilized for 2 d to afford an off-white solid.

Poly(**2a**)_50_: ^1^H NMR (700 MHz, D_2_O) δ 7.50–7.25 (m), 6.24 (br, s), 5.78 (br, s), 5.05 (br, s), 4.90 (br, s), 4.89–4.84 (m), 3.99 (br, s), 3.98–3.72 (m), 3.70–3.33 (m), 3.25 (br, s), 2.41–1.90 (m), 1.88–0.84 (m).

Poly(**2b**)_50_: ^1^H NMR (700 MHz, D_2_O) δ 7.45–7.24 (m), 6.27 (br, s), 4.35 (br, s), 3.92 (br, s), 3.80–3.75 (m), 3.74 (br, s), 3.65 (dd, *J* = 11.8, 4.4 Hz), 3.55 (dd, *J* = 11.6, 6.5 Hz), 3.49 (br, s), 2.75–1.90 (m), 1.88–1.32 (m), 1.30–1.16 (m).

Poly(**3a**)_50_: ^1^H NMR (700 MHz, D_2_O) δ 7.40–7.10 (m), 6.25 (br, s), 5.83 (br, s), 5.63 (br, s), 5.26 (br, s), 4.86 (br, s), 4.83 (br, s), 4.75 (br, s), 4.71 (br, s), 4.21 (br, s), 4.16–3.99 (m), 3.91 (br, s), 3.87–3.82 (m), 3.81–3.73 (m), 3.70–3.64 (m), 3.60 (br, s), 3.55–3.36 (m), 2.77 (br, s), 2.65 (br, s), 2.39 (br, s), 2.19 (br, s), 1.65 (br, s), 1.61–1.12 (m).

Poly(**3b**)_50_: ^1^H NMR (700 MHz, D_2_O) δ 7.27–7.13 (m), 6.25 (br, s), 5.94–5.62 (m), 5.27 (br, s), 5.07 (br, s), 4.40–4.32 (m), 4.31–3.99 (m), 3.90 (br, s), 3.81–3.69 (m), 3.67 (dd, *J* = 11.8, 4.5 Hz), 3.59 (t, *J* = 6.6 Hz), 3.55 (dd, *J* = 11.8, 6.5 Hz), 3.52–3.39 (m), 2.68 (br, s), 2.40 (br, s), 2.19 (br, s), 1.65 (br, s), 1.42 (br, s), 1.30–1.20 (m).

### Small-Angle X-ray Scattering (SAXS)

Experiments were conducted on the Life Science X-ray Scattering (LiX) beamline, 16-ID, at the National Synchrotron Light Source II (NSLS II) at Brookhaven National Laboratory in Upton, NY. Stock solutions of the deprotected glycopolymers were prepared at 1% (w/v) in M16 medium. Aliquots of the stock solutions (60 μL) were then pipetted into PCR tubes, placed in LiX holders, and measured using the automated data collection procedure at the beamline.^26^ SAXS and wide-angle X-ray scattering (WAXS) data were collected simultaneously on Pilatus 1 M (SAXS) and Pilatus 900 K (WAXS) detectors.^27^ Data collected from both detectors was then scaled and merged. Ten frames with 0.5 s exposure were averaged, and outliers were removed automatically. Buffer subtraction from the sample was normalized using the water peak height at 2.0 Å^–1^. Data processing and analysis were performed using the py4XS and lixtools Python scripts as well as the SasView software package.

## Results and Discussion

### Design and Preparation of [4.2.0] Glycopolymers

**Scheme 1:**
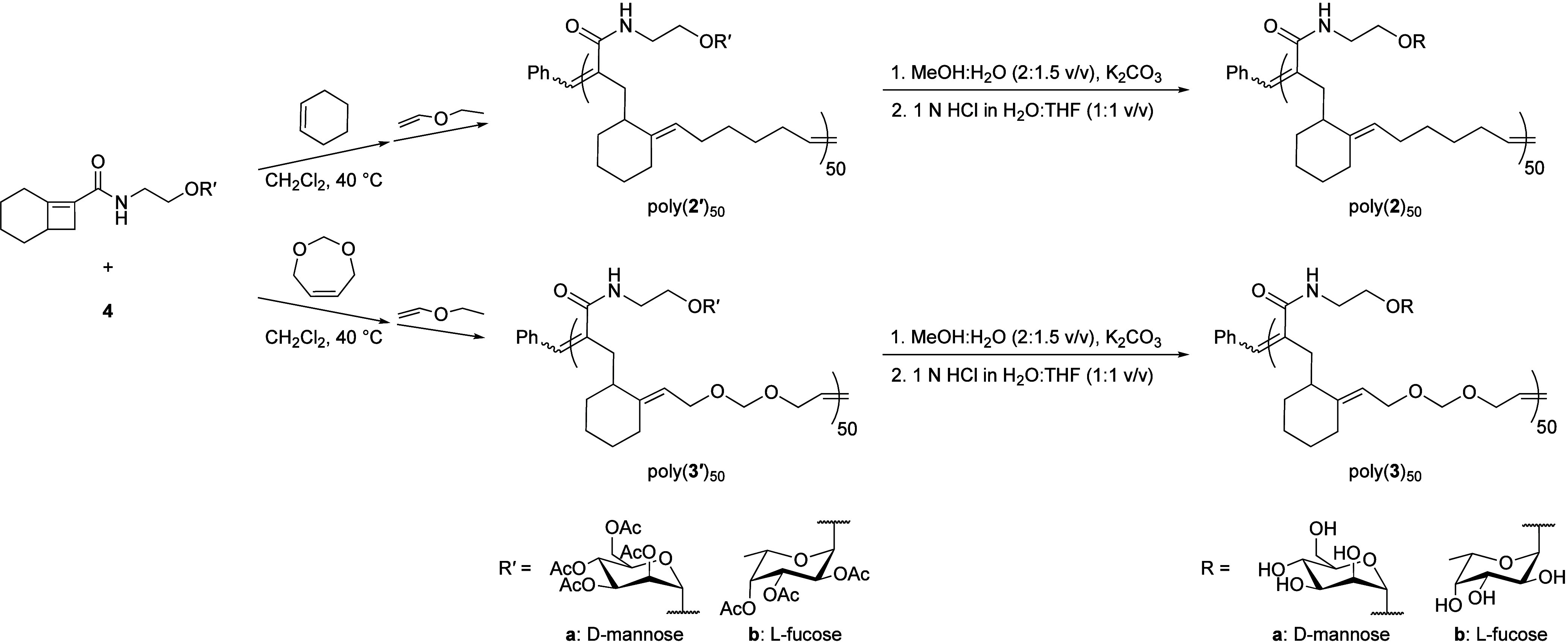
Synthesis of Alternating Glycocopolymers

The ruthenium-catalyzed AROMP of low-strain cyclic olefins and bicylo[4.2.0]octenes has been investigated extensively and has been championed for producing copolymers that can display a variety of functional groups, controlled backbone sequences, and tunable material properties such as hydrophilicity and glass transition temperatures.^7−10,28−30^ To prepare the [4.2.0] glycopolymers, 4,7-dihydro-1,3-dioxepin or cyclohexene (B monomer) was allowed to react with bicyclo[4.2.0]oct-6-ene-7-carboxamide bearing mannose or fucose (A monomer) in the presence of Grubbs’ third generation catalyst **4** (Scheme 1).

Despite previous accomplishments in synthesizing long, alternating AB copolymers with large molecular weights,^9,10^ the highest degree of polymerization that could be achieved for the [4.2.0] glycopolymers was 50 AB units. The presence of sugar moieties on bicyclo[4.2.0]oct-6-ene-7-carboxamide seemed to significantly lower the reactivity of the reaction, most likely due to steric hindrance as suggested by previous studies.^30,31^ Despite allowing the reaction to run for longer than 20 h, copolymers significantly longer than 50 AB units could not be achieved. We have previously demonstrated that polynorbornene 100-mers displaying mannose and fucose efficiently induce AE in mouse sperm^23−25^ and polynorbornene 50-mers display the same number of ligands as the [4.2.0] glycopolymers. Thus, we undertook comparison of the [4.2.0] glycopolymers to polynorbornene 100-mers and 50-mers in our AE activation experiments.

The purities of the acetylated polymers were confirmed by ^1^H NMR and ^13^C NMR spectroscopy. The number-average (*M*_n_) and weight-average (*M*_w_) molecular weights and dispersities (*Đ*_M_) were determined by GPC with polystyrene as a standard, which may underestimate molecular weights (Table 1). All of the glycopolymers had low to moderate molecular weight distributions between 1.1 and 1.7. Once characterized, the polymers were deacetylated, purities were determined by ^1^H NMR spectroscopy, and conformations of the structures in solution were determined by SAXS analysis (Tables 2 and 3).

**Table 1:** Average Molecular Weights and Dispersities of Poly(**1′**)_100_, Poly(**1′**)_50_, Poly(**2′**)_50_, and Poly(**3′**)_50_

polymer	*M*_n_^theor,^^a^	*M*_n_^b^	*M*_w_^b^	*Đ*_M_^b^
poly(**1a′**)_100_ (I)^c^	51,260	46,540	54,110	1.16
poly(**1a′**)_100_ (II)	51,260	44,220	52,320	1.18
poly(**1b′**)_100_ (I)	45,450	41,700	50,420	1.21
poly(**1b′**)_100_ (II)	45,450	40,340	49,320	1.22
poly(**1a′**)_50_ (I)	25,680	19,800	21,800	1.10
poly(**1a′**)_50_ (II)	25,680	5,270	7,247	1.38
poly(**1b′**)_50_ (I)	22,780	19,280	20,940	1.09
poly(**1b′**)_50_ (II)	22,780	5,616	7,018	1.25
poly(**2a′**)_50_ (I)	30,490	28,440	46,520	1.64
poly(**2a′**)_50_ (II)	30,490	29,210	36,990	1.27
poly(**2b′**)_50_ (I)	27,590	26,890	33,680	1.25
poly(**2b′**)_50_ (II)	27,590	21,060	35,660	1.69
poly(**3a′**)_50_ (I)	31,390	25,710	39,890	1.55
poly(**3a′**)_50_ (II)	31,390	27,450	46,340	1.69
poly(**3b′**)_50_ (I)	28,490	27,870	46,980	1.69
poly(**3b′**)_50_ (II)	28,490	14,890	24,650	1.66

a Theoretical molecular weights (*M*_n_^theor^) were calculated based on the catalyst-to-monomer ratio and 100% conversion of monomer.

b Values measured by GPC refractive index (RI) using polystyrene standards.

c (I) and (II) denote the polymer batch number.

**Table 2:** SAXS Data Fit Parameters for 1% (w/v) Poly(**1**)_100_ and Poly(**1**)_50_ Fit to a Flexible Cylinder Model

polymer	contour length, *L* (Å)	Kuhn length, 2*l*_p_ (Å)	radius, *R* (Å)
poly(**1a**)_100_ (I)^a^	396.8 ± 11.9	29.4 ± 1.7	8.3 ± 0.1
poly(**1b**)_100_ (II)	448.5 ± 10.9	46.3 ± 2.4	8.9 ± 0.1
poly(**1a**)_50_ (I)	348.3 ± 9.3	46.7 ± 2.3	8.8 ± 0.2
poly(**1b**)_50_ (II)	299.5 ± 0.9	90.4 ± 0.3	8.5 ± 0.03

a (I) and (II) denote the polymer batch number.

**Table 3:** SAXS Data Fit Parameters for 1% (w/v) Poly(**2**)_50_ and Poly(**3**)_50_ Fit to the Guinier–Porod Model

polymer	Porod exponent (*m*)^a^	*R*_g_ (Å)^a^	*s*^a^	power^b^
poly(**2a**)_50_ (II)^c^	3.8 ± 0.003	207.3 ± 1.4	1.0 ± 0.01	
poly(**2b**)_50_ (II)	3.7 ± 0.006	211.0 ± 3.7	1.2 ± 0.08	
poly(**3a**)_50_ (I)	1.5 ± 0.02	412.4 ± 33.3	1.1 ± 0.02	3.5 ± 0.05
poly(**3b**)_50_ (I)	1.9 ± 0.02	381.0 ± 30.8	1.1 ± 0.03	3.9 ± 0.03

a Parameters for the Guinier–Porod model.

b Parameter for the power law model. In the case of poly(**3a**)_50_ and poly(**3b**)_50_, a power law model was combined with the Guinier–Porod model.

c (I) and (II) denote the polymer batch number.

### Structural Analysis of Glycopolymer Backbones

To determine the structural characteristics of [4.2.0] glycopolymers, poly(**2**)_50_ and poly(**3**)_50_, the polymer conformations were quantified by SAXS analysis. For comparison, the solution structures of polynorbornene glycopolymers were determined. SAXS data were collected on 1% (w/v) deacetylated polymer solutions in M16 buffer. M16 was the same buffer utilized in biological assays; however, in the case of the SAXS experiments, the media was not supplemented with BSA to avoid interference from the X-ray scattering of this protein. M16 buffer was also used for the background signal of the SAXS experiments and subtracted from the data collected.

Data for all polymers was collected in a *q* range of 0.005 to 3.19 Å^–1^. However, poly(**1**)_100_, poly(**1**)_50_, and poly(**2**)_50_ samples were fit in a *q* range of 0.005–0.4 Å^–1^. This range was chosen because this is the relevant SAXS scattering window. Although error in the majority of the SAXS region is negligible (<1%), uncertainties are larger in the WAXS region, for example, *q* greater than 1.0 Å^–1^, where the scattering is dominated by the signal from water. Additionally, for the *q* range below 0.01 Å^–1^, the signal from the background scattering is very large, resulting in greater uncertainties after background subtraction in comparison to other regions. Due to low sample signal scattering in the 0.2–0.4 Å^–1^ range, poly(**3**)_50_ samples were fit in a *q* range of 0.005 to 0.22 Å^–1^. Subtracted scattering data from samples was fit to a flexible cylinder model or a Guinier–Porod model combined with a power law model using SasView. The error for each fit parameter was determined in SasView through its effect on the chi-squared value for the fit.

### Structural Analysis of Poly(**1**)_100_ and Poly(**1**)_50_

**Figure 2:**
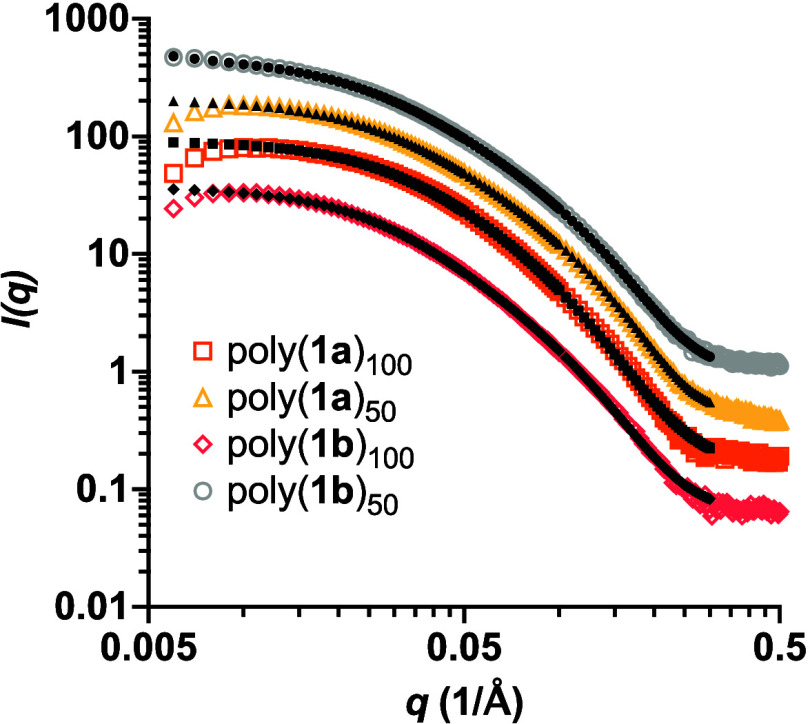
SAXS data plots of poly(**1**)_100_ and poly(**1**)_50_ at 1% (w/v) in M16 buffer. The glycopolymers were fit to a flexible cylinder model. Fits encompassed data where the scattering is above background. Each data set was offset by an arbitrary amount for clarity. The color traces represent the glycopolymer data, whereas the black traces correspond to the fits of the data.

**Figure 3:**
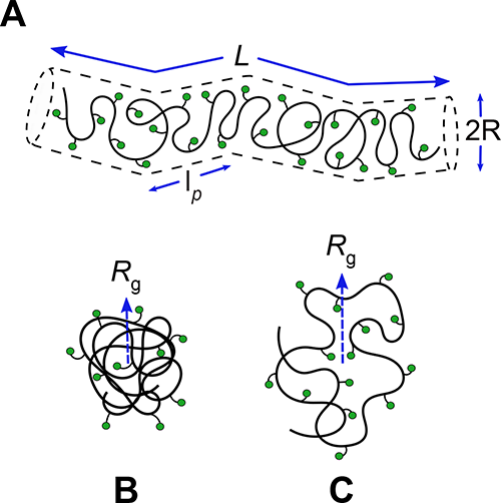
Proposed solution structures consistent with the SAXS models of poly(**1**)_100_, poly(**1**)_50_, poly(**2**)_50_, and poly(**3**)_50_. (A) Poly(**1**)_100_ and poly(**1**)_50_ adopt a flexible cylinder conformation where *L* is the contour length, *R* is the radius, and 2*l*_p_ is the Kuhn length. (B) Poly(**2**)_50_ assumes a compact structure due to collapsed polymer chains in a poor solvent despite possessing rod-like characteristics. (C) Poly(**3**)_50_ adopts a rod-like yet flexible conformation seen in swollen polymer chains in a good solvent. The radius of gyration, *R*_g_, is denoted by a blue dashed arrow.

In Figure 2, scattering data and model fits for polynorbornene samples of either 50 or 100 repeating units of the fucose or mannose sugars are shown. The slope at low-*q* approaches 0 for all of the polymers, indicating that there are defined free-floating scattering particles in solution. Based on the slope of the curves, poly(**1**)_100_ and poly(**1**)_50_ were fit to a flexible cylinder model (Figure 3A), which is consistent with our previously reported data on norbornene homopolymers,^25^ and the fits for polymers with a norbornene backbone were performed by others.^32−34^ The flexible cylinder model contains the physical parameters: length, Kuhn length, and radius. The length parameter is the contour length, *L*, of the chain or network. The radius, *R*, describes the circular face of the cylinder, while the Kuhn length, 2*l*_p_, is the length of a segment or link in the long polymer chain.^35^ Dispersity of the radius and Kuhn length was added as a Gaussian distribution about the parameter value.^36^ The results for each norbornene glycopolymer are summarized in Table 2.

Based on the fits, the norbornene polymers all had very similar radii ranging from 0.8 to 0.9 nm. In addition, the lengths of both 50-mers appeared to be similar to poly(**1a**)_50_ possessing a contour length of 34.8 ± 0.9 nm and poly(**1b**)_50_ with a contour length of 29.9 ± 0.09 nm. The 100-mers, poly(**1a**)_100_ and poly(**1b**)_100_, had contour lengths that were larger than the 50-mers, as expected. Interestingly, the contour lengths of the 100-mers were not double the size of the 50-mers but only 1.1 to 1.5 times longer. This suggests that the glycopolymers may be adopting a folded or coiled conformation. This coiled conformation is further supported by the Kratky plots of poly(**1**)_100_ and poly(**1**)_50_, which provide evidence of partially unfolded structures (Figure S2). Assuming the length of a polynorbornene monomer unit is 0.62 nm,^37^ a fully extended 50-mer or 100-mer polymer chain would have a contour length of approximately 31.0 or 62.0 nm, respectively. However, it is unlikely that this class of polymer would remain uncoiled in a solvent like M16 because of a combination of hydrophobic interactions with the solvent and favorable inter- and intramolecular interactions caused by the presence of amides and hydroxyl groups in the polymer chain. Therefore, the contour lengths could be due to interlinking or entangled chains of irregularly coiled polymers.

The Kuhn lengths of poly(**1**)_50_ suggested that there were long, rigid segments within the polymer chains. Poly(**1a**)_50_ and poly(**1b**)_50_ each had a Kuhn length of 4.7 ± 0.2 and 9.0 ± 0.03 nm, respectively. Surprisingly, the Kuhn lengths of poly(**1a**)_100_ and poly(**1b**)_100_ dropped significantly to 2.9 ± 0.2 and 4.6 ± 0.2 nm, respectively. The shorter Kuhn lengths present in the 100-mers are likely due to the 100-mers adopting a tighter coiled conformation. Tighter coiling could result in shorter segments, whereas the norbornene 50-mers are less packed and have longer segments. Moreover, the Kuhn lengths are dependent on the hydrophobicity of the sugar, with the more hydrophobic fucose resulting in a longer Kuhn length for poly(**1b**). Overall, the data suggest that polynorbornene 100-mers are longer than the 50-mers with a more tightly folded conformation.

### Structural Analysis of Poly(**2**)_50_ and Poly(**3**)_50_

When comparing the scattering of the polynorbornene polymers to poly(**2**)_50_ and poly(**3**)_50_, some fundamental differences were observed. There was a change in the slope at low-*q* for both poly(**2**)_50_ and poly(**3**)_50_, suggesting that the [4.2.0] glycopolymers adopted different conformations than polynorbornene. For this reason, the Guinier–Porod model was implemented to fit the poly(**2**)_50_ and poly(**3**)_50_ SAXS data. The Guinier–Porod model is an empirical model utilized for materials with arbitrary shapes and can provide general properties of the conformation of a structure in solution, especially for nonspherical objects. The fitting parameters of the Guinier–Porod model are the radius of gyration (*R*_g_), the Porod exponent (*m*), and a dimensionality parameter (*s*).^38^ The radius of gyration provides an estimate of the size of the particles, the Porod exponent correlates to the orientation or shape of the particles, and the dimensionality parameter describes the elongation of scattering particles where values close to 0 represent spheres or globules, 1 corresponds to rod-like objects, and 2 represents discs or platelets. In the case of poly(**3**)_50_, this model was combined with a power law model, which contains parameters for scale and power (eqs S3–S8).

**Figure 4:**
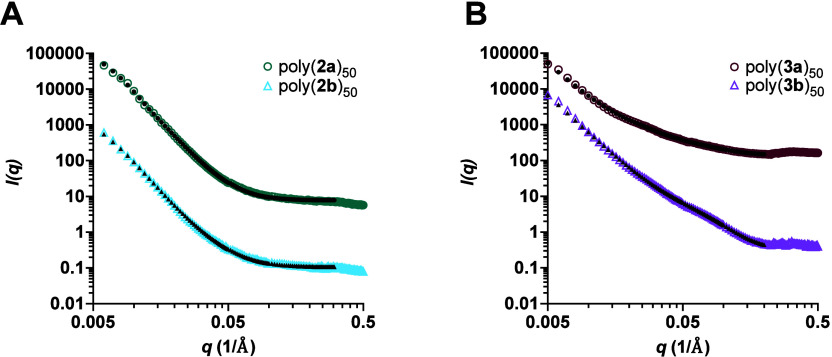
SAXS data plots of (A) poly(**2**)_50_ and (B) poly(**3**)_50_ at 1% (w/v) in M16 buffer. The glycopolymers were fit to a Guinier–Porod model. Fits encompassed data where the scattering is above background. Each data set was offset by an arbitrary amount for clarity. The color traces represent the glycopolymer data, whereas the black traces correspond to the fits to the data.

The scattering data from the glycopolymers with a [4.2.0]-cyclohexene backbone are shown in Figure 4A, and the SAXS results for poly(**2**)_50_ are summarized in Table 3. The slope of the data was used to determine the Porod exponents of each polymer and ascertain information about polymer flexibility. A Porod exponent between 3 and 4 indicates a surface fractal. More specifically, a Porod exponent of 3 suggests a very rough surface from collapsed polymer chains or rods in a bad solvent while an exponent of 4 points to particles with a smooth surface, such as a sphere.^38^ The Porod exponents for poly(**2a**)_50_ and poly(**2b**)_50_ were 3.8 and 3.7, respectively, indicating globular or compact structures. This is likely due to the increased flexibility of these polymers; the linear, unbranched alkyl segments in the backbones allow for increased bond rotation. As a result, to minimize hydrophobic interactions in an aqueous solvent like M16, the polymers readily collapse, leading to smooth, compact structures. Interestingly, *s* was very close to 1 for poly(**2**)_50_, indicating that the scatterers are rod-like. So, while the overall shapes of the particles were rod-like, the packing due to polymer collapse or aggregation in a poor solvent could be the reason for an increase in the Porod exponent. Finally, the *R*_g_ for poly(**2a**)_50_ was 20.7 ± 0.1 and 21.1 ± 0.4 nm for poly(**2b**)_50_. These values are more than twice the size of the radii observed for poly(**1**)_100_ and poly(**1**)_50_, which is consistent with poly(**2**)_50_ adopting a more compact structure and aggregating in a poor solvent (Figure 3B).

The scattering from glycopolymers with a [4.2.0]-dioxepin backbone is presented in Figure 4B. The results of the SAXS experiments are summarized in Table 3. The background for these samples was large compared to the scattering of the polymers, and, in some cases, the background was greater. This phenomenon can be seen as oversubtraction leading to negative scattering at higher *q*. Additionally, there is a change in the slope at low *q*, which is possibly an error resulting from the background subtraction. These effects make the scattering patterns more difficult to quantitatively analyze, but some information can still be obtained. For these reasons, the fitting parameters for these polymers tended to have more error and reduced certainty. To improve the fit, a power law was added to the Guinier–Porod model. This can account for additional scattering at low *q*. The window for the fitting was also reduced to only using the positive scattering below 0.22 Å^–1^. When analyzing the power law slope for poly(**3**)_50_, each sample had a value close to the power law dependence of the M16 medium, 3.4 ± 0.01, which supports the belief that the scattering was coming from the background.

In the case of poly(**3**)_50_, the *s* parameters were comparable to poly(**2**)_50_ in that they were close to 1, indicating that these are rod-like polymers. However, the Porod exponents for poly(**3**)_50_ were much lower than that of poly(**2**)_50_; the Porod exponents for poly(**3a**)_50_ and poly(**3b**)_50_ were 1.5 and 1.9, respectively. A Porod exponent of 1.67 corresponds to the scattering from fully swollen chains in a good solvent and an exponent of 2 points to scattering from Gaussian polymer chains or a two-dimensional structure.^38^ The *s* parameter and lower Porod exponents indicate that these samples are much less compact than the poly(**2**)_50_ glycopolymers. This is likely due to the presence of the additional oxygen atoms in the polymer backbone; the hydrophilic backbone of poly(**3**)_50_ allows for favorable interactions with the solvent, resulting in increased polymer stability and exposure. The *R*_g_ values further support this theory as poly(**3a**)_50_ had an *R*_g_ of 41.2 ± 3.3 nm and poly(**3b**)_50_ had an *R*_g_ of 38.1 ± 3.1 nm. These radii are nearly double the radii of poly(**2**)_50_, which is consistent with poly(**3**)_50_ possessing a less compact structure due to its hydrophilic properties. However, a less compact average orientation would result in a more flexible backbone in solution with less rigidity than poly(**1**)_100_, poly(**1**)_50_, and poly(**2**)_50_ (Figure 3C).

### Applications of [4.2.0] Glycopolymers in Acrosomal Exocytosis

The purpose of the [4.2.0] glycopolymers was to introduce new functionality to our previously established alternating copolymers and expand the applications of these structures, specifically, their biological relevance. In this case, we tested [4.2.0] glycopolymers displaying mannose and fucose ligands as inducers of AE in mouse sperm and determined how the structural effects of the glycopolymers influenced activation. Although the exact signaling pathways remain unknown, spermatozoa undergo a series of binding interactions with glycoproteins of the egg cell’s zona pellucida (ZP). In particular, terminal sugar moieties located on the ZP have been shown to play an important role in the interaction between sperm and the egg, contributing to the induction of AE.^13,39,40^ Previous studies in our laboratory demonstrated that synthetic glycopolymers, synthesized by ruthenium-catalyzed ring-opening metathesis polymerization (ROMP), were able to mimic physiological inducers and activated AE in mouse sperm in a dose-dependent manner.^23^ In a later study, induction of AE with glycopolymers consisting of norbornene or cyclooctene backbones revealed the requirements of polymer rigidity for AE activation.^25^ Therefore, polynorbornene 100-mers comprising mannose or fucose moieties were utilized as positive controls to examine the efficacy of the [4.2.0] glycopolymers in inducing AE. There is contradicting evidence suggesting that AE induction with ionophores like A23187 may or may not be a predictor of fertilization ability.^41−47^ We have previously demonstrated that AE induction with these glycopolymers produces comparable AE% levels to A23187.^23−25^ As a negative control, mouse sperm were treated with Dulbecco’s phosphate-buffered saline (DPBS) resulting in only 7.2% of live sperm undergoing spontaneous AE. Because the degree of polymerization of [4.2.0] glycopolymers was limited to 50 AB units, polynorbornene 50-mers were also included as a direct comparison for the number of ligands present on the [4.2.0] glycopolymers.

Using a triple-stain flow cytometry assay, capacitated mouse sperm were initially treated with each glycopolymer to determine the efficacy of AE induction at polymer concentrations 0.1, 1, 5, 10, and 20 μM. Polymer concentrations above 20 μM were not tested for any of the glycopolymers because a decrease in AE activity and sperm viability was observed at higher polymer concentrations. In addition to AE activity, we utilized our flow cytometry assay to monitor the viability of mouse sperm after treatment with glycopolymers. At each polymer concentration, the percentage of live cells (PI negative) was recorded and averaged. There was no statistically significant difference in cell viability between samples treated with the negative control or the glycopolymers (Figure S3) confirming that poly(**2**)_50_ and poly(**3**)_50_ are not cytotoxic at the concentrations used in the biological assay.

### Effects of Poly(**1**)_100_ and Poly(**1**)_50_ on AE Induction in Mouse Sperm

**Figure 5:**
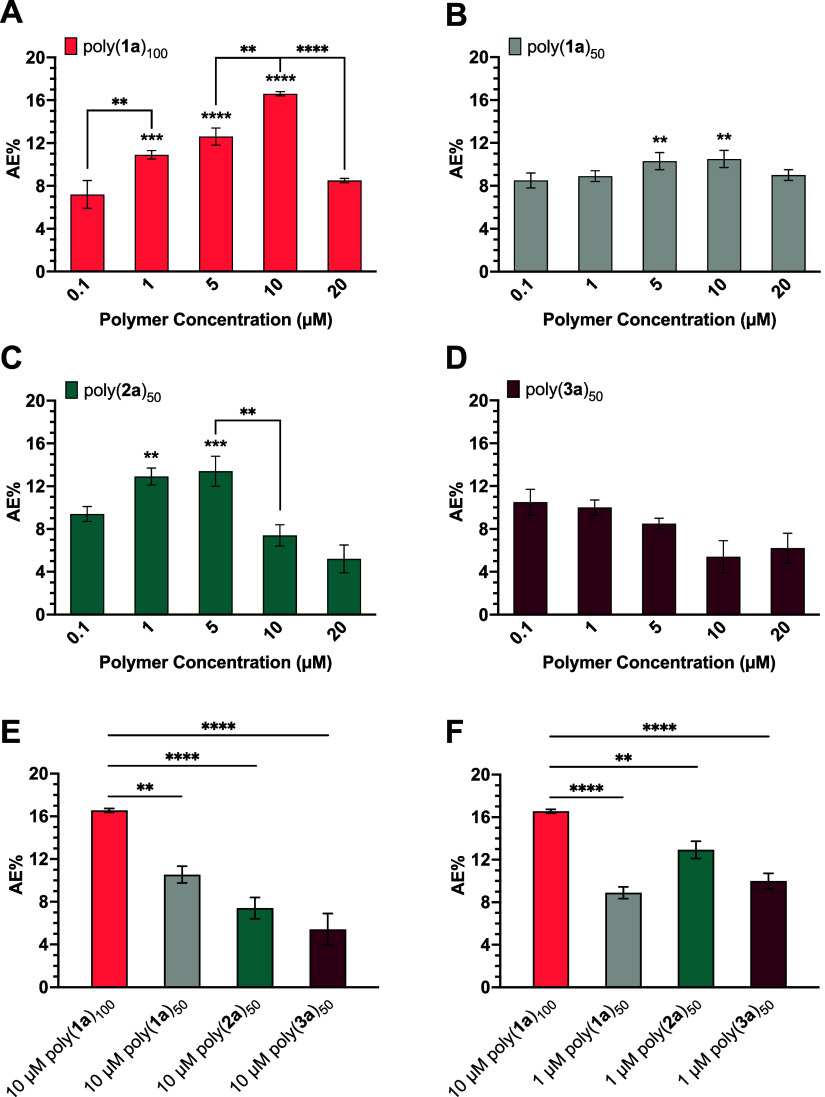
AE activation in mouse sperm by (A) poly(**1a**)_100_, (B) poly(**1a**)_50_, (C) poly(**2a**)_50_, and (D) poly(**3a**)_50_. The AE% of glycopolymers at (E) 10 and (F) 1 μM was compared to the maximum AE% of poly(**1a**)_100_. The average AE% for mouse sperm treated with DPBS (negative control) was 7.2%. Data represents mean ± standard error of the mean of at least three independent experiments testing two batches of each polymer. One-way ANOVA was used to compare AE% of glycopolymer induction to DPBS, AE% of consecutive polymer concentrations, and AE% of glycopolymers at 1 and 10 μM. *p < 0.05, **p < 0.01, ***p < 0.001, ****p < 0.0001 for all comparisons.

**Figure 6:**
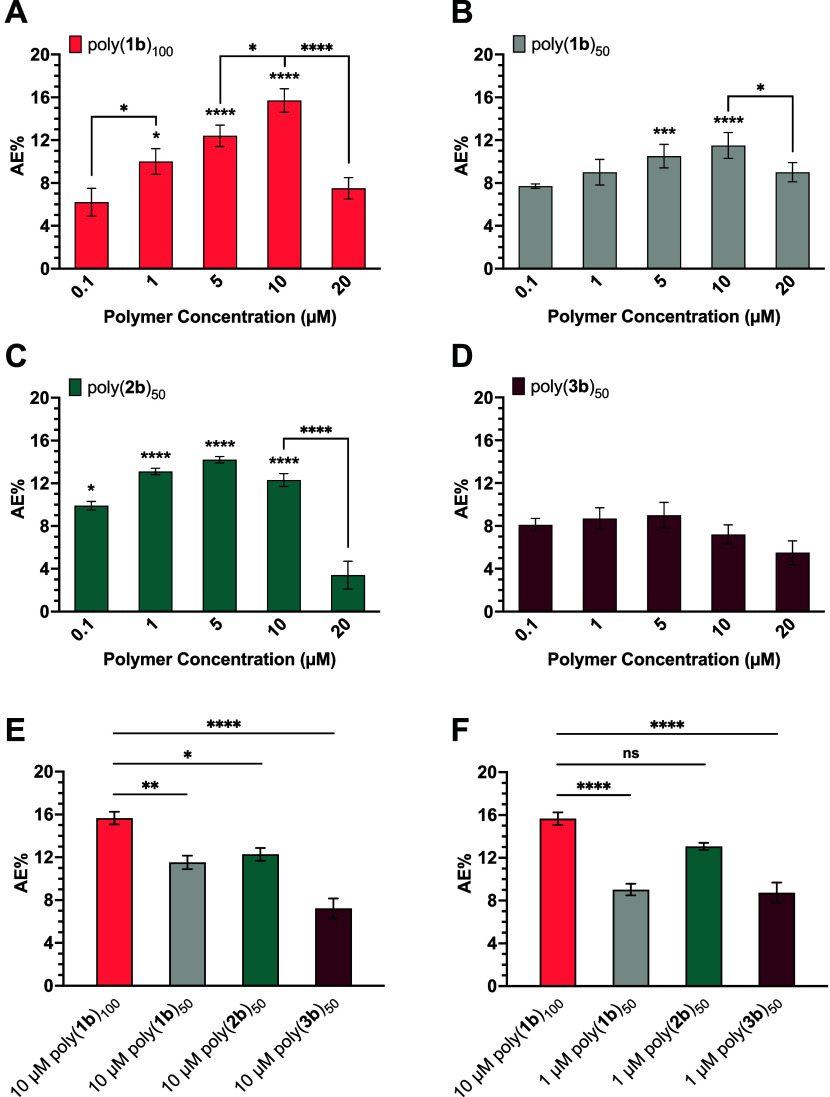
AE activation in mouse sperm by (A) poly(**1b**)_100_, (B) poly(**1b**)_50_, (C) poly(**2b**)_50_, and (D) poly(**3b**)_50_. The AE% of glycopolymers at (E) 10 and (F) 1 μM were compared to the maximum AE% of poly(**1b**)_100_. The average AE% for mouse sperm treated with DPBS (negative control) was 7.2%. Data represents mean ± standard error of the mean of at least three independent experiments testing two batches of each polymer. One-way ANOVA was used to compare AE% of glycopolymer induction to DPBS, AE% of consecutive polymer concentrations, and AE% of glycopolymers at 1 and 10 μM. *p < 0.05, **p < 0.01, ***p < 0.001, ****p < 0.0001 for all comparisons.

Polynorbornene 100-mers displaying fucose and mannose were effective at inducing AE in a dose-dependent manner; as the concentration of polymer increased, an increase in AE% was observed. Treatment of mouse sperm with 10 μM of poly(**1a**)_100_ or poly(**1b**)_100_ resulted in a maximum AE% of ∼17% (Figures 5A and 6A), which aligned with our previous studies,^23−25^ and the levels of AE expected for a typical mouse sperm sample. Polynorbornene 50-mers displaying either fucose or mannose seemed to activate AE in mouse sperm (Figures 5B and 6B). However, it was evident that the efficacy of AE induction decreased significantly once the norbornyl polymer chains were shortened from 100-mers to 50-mers; at 10 μM, the maximum AE% observed decreased by approximately 1.6-fold and 1.4-fold when the norbornyl polymers were shortened to poly(**1a**)_50_ and poly(**1b**)_50_, respectively (Figures 5E and 6E). These results correlate with the SAXS data analysis as poly(**1**)_100_ was shown to have slightly longer contour lengths than poly(**1**)_50_. Thus, the results of this work align with our previous findings suggesting the optimal length and rigidity of poly(**1**)_100_ are necessary for maximal AE induction in mouse sperm.^23,25,48^

### Effects of Poly(**2**)_50_ on AE Induction in Mouse Sperm

Poly(**2a**)_50_ and poly(**2b**)_50_ possessed all-carbon, hydrophobic backbones like poly(**1**)_100_ albeit with very different structures. Like the polynorbornene 100-mers, poly(**2**)_50_ activated AE in mouse sperm (Figures 5C and 6C). Interestingly, 10 μM poly(**1a**)_100_ or poly(**1b**)_100_ was equivalent in AE-inducing activity to 1 μM of poly(**2a**)_50_ or poly(**2b**)_50_. That is, 10-fold less polymer concentration was needed to achieve comparable AE results (Figures 5F and 6F). We attribute this difference in AE activation efficacy to the structure of the [4.2.0]-cyclohexene polymers. Our previous study demonstrated that a spherical, compact structure could induce AE in mouse sperm if it was paired with mannose ligands.^25^ The [4.2.0]-cyclohexene polymers in these experiments possess *R*_g_ values and Porod exponents that are very similar to the spherical conformation of the cyclooctene glycopolymer that induced AE. We suggest that the densely compacted, smooth nature of poly(**2**)_50_ provides sufficient polymer rigidity to induce AE when paired with an activating sugar, supporting our hypothesis that polymer rigidity is necessary for activation.

In addition, the AE activation profile of poly(**2**)_50_ shifts to lower concentrations compared to the AE activation profile of poly(**1**)_100_. We expect that at higher concentrations like those used to obtain SAXS data, poly(**2**)_50_ forms compact structures as there is greater potential for intermolecular interactions among the polymer side chains. Conversely, lower concentrations of poly(**2**)_50_, which were used in the biological assays, will maximize AE induction as the polymer–polymer interactions would no longer drive the formation of compact structures, and an increased number of ligands will be available to interact with receptors on the sperm. The concept of polymer conformation and rigidity changing based on polymer concentration has been studied in bottlebrush polymers possessing polynorbornene and poly(2-isopropenyl-2-oxazoline) backbones in good solvents.^49,50^ In both cases, increasing the concentration of polymer resulted in an overall decrease in polymer stiffness, and ultimately, a reduction in the *R*_g_ of the polymers as polymer–polymer interactions dominated polymer–solvent interactions at high concentrations. A similar phenomenon may be occurring with the semirigid structures of our polymers and could explain why AE activity decreases as polymer concentration increases. For the case of poly(**2**)_50_, this transition may occur at lower concentrations than for poly(**1**)_100_.

### Effects of Poly(**3**)_50_ on AE Induction in Mouse Sperm

The backbones of poly(**3**)_50_ are more hydrophilic than the backbones of poly(**2**)_50_ due to the presence of two oxygen atoms. Modifications to the hydrogen-bonding properties of these polymers allowed for further investigation of the importance of polymer backbone hydrophobicity on glycopolymer-induced AE. Interestingly, the addition of heteroatoms to the polymer backbone resulted in little to no AE induction (Figures 5D and 6D). As demonstrated in the SAXS data analysis, poly(**3a**)_50_ and poly(**3b**)_50_ possess larger *R*_g_ values, suggesting that the polymers are much less compact and favorably interact with the solvent. As a result, these structures are more flexible and, therefore, are ineffective at inducing AE despite presenting the same density of sugars as poly(**2**)_50_. Thus, polymer rigidity appears to play a dominant role in activation, further corroborating our previous study.^25^

## Conclusions

Alternating copolymers comprised of bicyclo[4.2.0]oct-6-ene-7-carboxamide bearing a mannose or fucose moiety and 4,7-dihydro-1,3-dioxepin or cyclohexene produced a new series of glycopolymers that can be used to probe biological processes mediated by receptor–ligand interactions and multivalency. Although the greatest degree of polymerization that could be achieved for these glycopolymers was 50 AB units, in terms of number of segments, these polymers are analogous to polynorbornene 100-mers. The alternating glycopolymers adopted distinct structural conformations depending on the hydrophilicity of the B monomer utilized. The hydrophilic backbone of the [4.2.0]-dioxepin glycopolymers provided swollen polymer chains with rod-like, flexible structures in aqueous isotonic media. Conversely, the hydrophobic backbone of the [4.2.0]-cyclohexene glycopolymers provided rod-like compact structures. These structural differences correlated with differential activities in biological assays for induction of AE in mouse sperm. Overall, we have developed two sets of [4.2.0] glycopolymers with distinct ligand spacing, hydrophilicity, and structural conformations. These glycopolymers can be utilized for the investigation of a variety of biological systems or processes based on the structural properties desired in a cell-binding probe.

## Supporting Information Available

Additional information on SAXS analysis and glycopolymer AE% comparisons; reaction schemes, NMR spectra, and SBTI-Alexa 488 conjugate and poly(**1**)_50_ synthesis, purification, and characterization (PDF)
