# Supplementary material for: Glycopolymers Prepared by Alternating Ring-Opening Metathesis Polymerization Provide Access to Distinct, Multivalent Structures for the Probing of Biological Activity
Source: ACS Bio Med Chem Au. 2024 May 28;4(4):214–25. doi: 10.1021/acsbiomedchemau.4c00018 (PMC11342347; doi:10.1021/acsbiomedchemau.4c00018)
Supplement: Supplementary file 1 — bg4c00018_si_001.pdf [file bg4c00018_si_001.pdf]

Supporting Information

Glycopolymers Prepared by Alternating Ring-Opening Metathesis Polymerization Provide Access to Distinct, Multivalent Structures for the Probing of Biological Activity

Luz C. Mendez[1], Francis O. Boadi[1], Mitchell Kennedy[1], Surita R. Bhatia[1], and Nicole S. Sampson[1,2,*]

[1]Department of Chemistry, Stony Brook University, Stony Brook, NY 11794-3400, United States

[2]Department of Chemistry, University of Rochester, Rochester, NY 14627-0216, United States

*Corresponding author: nicole.sampson@rochester.edu

Table of Contents

Preparation of SBTI-Alexa 488 conjugate S4

UV-vis analysis of SBTI-Alexa 488 conjugate S4

Equation S1 and Equation S2 to calculate the concentration of protein conjugate and degree of labeling. S5

Equations S3–S8. Guinier-Porod model and power law equations S6

General methods S7

General preparation of poly(**1′**)$_{100}$ and poly(**1**)$_{100}$ S7

General preparation of poly(**1′**)$_{50}$ S7

General preparation of poly(**1**)$_{50}$ S8

Scheme S1. Synthesis of poly(**1′**)$_{50}$ and poly(**1**)$_{50}$ S8

Scheme S2. Synthesis of 2,3,4,6-tetra-O-acetyl-α-D-mannopyranosyl bicyclo[4.2.0]oct-6-ene-7-carboxamide S9

Scheme S3. Synthesis of 2,3,4-tri-O-acetyl-L-fucopyranosyl bicyclo[4.2.0]oct-6-ene-7-carboxamide S9

Table S1. Protein concentrations and degree of labeling for SBTI-Alexa 488 conjugates prepared S10

Figure S1. UV-vis traces of SBTI protein, Alexa Fluor™ 488 dye, and SBTI-Alexa 488 conjugate S11

Figure S2. Kratky plots of poly(**1**)$_{100}$ and poly(**1**)$_{50}$ S12

Figure S3. Comparison of the average percent of live mouse sperm after treatment with glycopolymers or the negative control (DPBS) S13

Figure S4. Comparison of AE induction in mouse sperm by mannose or fucose glycopolymers S14

Figure S5. ^{1}H NMR (700 MHz, $CDCl_3$) spectrum of poly(**1a′**)$_{50}$ S15

Figure S6. ^{13}C NMR (176 MHz, $CDCl_3$) spectrum of poly(**1a′**)$_{50}$ S16

Figure S7. ^{1}H NMR (400 MHz, D_2O) spectrum of poly(**1a**)$_{50}$ S17

Figure S8. ^{1}H NMR (700 MHz, $CDCl_3$) spectrum of poly(**1b′**)$_{50}$ S18

Figure S9. ^{13}C NMR (176 MHz, $CDCl_3$) spectrum of poly(**1b′**)$_{50}$ S19

Figure S10. ^{1}H NMR (400 MHz, D_2O) spectrum of poly(**1b**)$_{50}$ S20

Figure S11. ^{1}H NMR (700 MHz, $CDCl_3$) spectrum of 2,3,4,6-tetra-O-acetyl-α-D-mannopyranosyl bicyclo[4.2.0]oct-6-ene-7-carboxamide S21

Figure S12. ^{13}C NMR (176 MHz, $CDCl_3$) spectrum of 2,3,4,6-tetra-O-acetyl-α-D-mannopyranosyl bicyclo[4.2.0]oct-6-ene-7-carboxamide S22

Figure S13. HRMS spectrum of 2,3,4,6-tetra-O-acetyl-α-D-mannopyranosyl bicyclo[4.2.0]oct-6-ene-7-carboxamide S23

Figure S14. ^{1}H NMR (700 MHz, $CDCl_3$) spectrum of 2,3,4-tri-O-acetyl-L-fucopyranosyl bicyclo[4.2.0]oct-6-ene-7-carboxamide S24

Figure S15. ^{13}C NMR (176 MHz, $CDCl_3$) spectrum of 2,3,4-tri-O-acetyl-L-fucopyranosyl bicyclo[4.2.0]oct-6-ene-7-carboxamide S25
Figure S16. HRMS spectrum of 2,3,4-tri-O-acetyl-L-fucopyranosyl bicyclo[4.2.0]oct-6-ene-7-carboxamide S26
Figure S17. ^{1}H NMR (500 MHz, CD_2Cl_2) spectrum of poly(**2a′**)$_{50}$ S27
Figure S18. ^{13}C NMR (125 MHz, CD_2Cl_2) spectrum of poly(**2a′**)$_{50}$ S28
Figure S19. ^{1}H NMR (700 MHz, D_2O) spectrum of poly(**2a**)$_{50}$ S29
Figure S20. ^{1}H NMR (700 MHz, CD_2Cl_2) spectrum of poly(**2b′**)$_{50}$ S30
Figure S21. ^{13}C NMR (176 MHz, CD_2Cl_2) spectrum of poly(**2b′**)$_{50}$ S31
Figure S22. ^{1}H NMR (700 MHz, D_2O) spectrum of poly(**2b**)$_{50}$ S32
Figure S23. ^{1}H NMR (700 MHz, CD_2Cl_2) spectrum of poly(**3a′**)$_{50}$ S33
Figure S24. ^{13}C NMR (176 MHz, CD_2Cl_2) spectrum of poly(**3a′**)$_{50}$ S34
Figure S25. ^{1}H NMR (700 MHz, D_2O) spectrum of poly(**3a**)$_{50}$ S35
Figure S26. ^{1}H NMR (700 MHz, CD_2Cl_2) spectrum of poly(**3b′**)$_{50}$ S36
Figure S27. ^{13}C NMR (176 MHz, CD_2Cl_2) spectrum of poly(**3b′**)$_{50}$ S37
Figure S28. ^{1}H NMR (700 MHz, D_2O) spectrum of poly(**3b**)$_{50}$ S38
References S39

Preparation of SBTI-Alexa 488 Conjugate. A 2 mg/mL solution of soybean trypsin inhibitor (SBTI) was prepared using 0.1 M $NaHCO_3$ (pH: 8.3). 500.0 µL (1.0 mg) of the protein solution was then transferred into a reaction vial containing Alexa Fluor™ 488 tetrafluorophenyl (TFP) ester that was warmed to 25 °C. The mixture was allowed to stir gently at 25 °C in the dark for 1 h. The reaction mixture was purified using size exclusion chromatography (Cytiva disposable PD-10 desalting column with Sephadex G-25 resin, 1.0–2.5 mL samples) with 1X Dulbecco's phosphate-buffered saline (DPBS, containing 0.02% sodium azide) as the elution buffer. The bottom yellow-green band in the column corresponding to the protein conjugate was collected. The top green band, which contained unreacted and hydrolyzed dye, was disposed. After purification, the protein conjugate was transferred to a prewetted Amicon® Ultra-15 Centrifugal Filter Unit (MWCO 3kD, 15 mL sample) and centrifuged at 5,000 rpm for 5 min to concentrate the protein conjugate to 1.0 mL. Once concentrated, the conjugate was prepared for relabeling. Two reaction vials of Alexa Fluor™ 488 TFP ester were warmed to 25 °C and combined in a single vial using 1.0 mL of protein conjugate. 100.0 µL of 0.1 M $NaHCO_3$ was then added to the reaction to increase the pH of the solution. The reaction mixture was then left to stir at 25 °C in the dark for 1 h before transferring to 4 °C and allowing to stir gently for an additional 24 h in the dark. The relabeled protein conjugate was then purified by size exclusion chromatography for a second time with 1X DPBS buffer (containing 0.02% sodium azide) as the elution buffer and analyzed by UV-vis spectroscopy.

UV-vis Analysis of SBTI-Alexa 488 Conjugate. UV-vis analysis of the SBTI-Alexa 488 conjugate was performed on a Shimadzu UV-vis spectrophotometer (UV-2550). 1X DPBS (containing 0.02% of sodium azide) was used as a reference and all of the readings were taken at 25 °C. Appropriate dilutions of the SBTI-Alexa 488 conjugate were made with 1X DPBS to ensure that the absorbance values were less than 1.0. Absorbance was measured in a black quartz cuvette with a 1 cm path length at 280 nm (A_{280}) and 494 nm (A_{494}) to account for the absorbance maximum of the Alexa Fluor™ 488 dye (Figure S1). The concentration of the labeled protein and the degree of labeling were calculated. The molar extinction coefficient of Alexa Fluor™ 488, correction factor of Alexa Fluor™ 488 emission at 280 nm, and the molecular weight of SBTI were all incorporated into the calculations (Equation S1 and Equation S2). The degree of labeling for various batches of protein conjugates ranged between 1 and 3 moles of dye per mole of protein, which was sufficient for our experiments (Table S1).

$$Protein\ concentration\ (M) = \frac{[A_{280} - 0.11(A_{494})] * dilution\ factor}{\varepsilon_{protein}}$$

Equation S1. Equation to calculate the concentration of labeled protein in molarity (M). A_{280} and A_{494} are the absorbances of SBTI-Alexa 488 at 280 nm and 494 nm, respectively. The molar extinction coefficient (ε) of the SBTI protein is approximately 20,000 $M^{-1}cm^{-1}$. 0.11 is the correction factor used to account for the absorbance emitted by the Alexa Fluor™ 488 dye at 280 nm.[1]

$$Moles\ of\ dye\ per\ mole\ of\ protein = \frac{A_{494} * dilution\ factor}{71{,}000 * protein\ concentration\ (M)}$$

Equation S2. Equation to calculate the degree of labeling (moles of Alexa Fluor™ 488 dye per mole of SBTI protein). A_{494} is the absorbance of SBTI-Alexa 488 at 494 nm. 71,000 $M^{-1}cm^{-1}$ is the approximate molar extinction coefficient of the Alexa Fluor™ 488 dye.[1]

Guinier-Porod Model and Power Law Equations. A Guinier-Porod model[5] was used for fitting data where the intensity is calculated as a piecewise function based on the scattering vector q_1 (Equation S3). Below q_1, scattering is dominated by Guinier scattering (Equation S4), and above q_1, scattering is defined by Porod scattering (Equation S5). The fitting parameters are the radius of gyration (R_g), the Porod exponent (m), a dimensionality parameter (s), and two scale factors: Guinier (G) and Porod (D) (Equation S6).

$$q_1 = \frac{1}{R_g}\sqrt{\frac{(m-s)(3-s)}{2}} \quad \text{(Equation S3)}$$

$$I(q) = scale * \frac{G}{q^s}\exp\left[\frac{-q^2 R_g^2}{3-s}\right] + background \quad \text{(Equation S4)}$$

$$I(q) = scale * \frac{D}{q^m} + background \quad \text{(Equation S5)}$$

$$D = G\exp\left[\frac{-q_1^2 * R_g^2}{3-s}\right] q_1^{m-s} \quad \text{(Equation S6)}$$

$$R_g = \frac{L}{12} + \frac{r^2}{2} \quad \text{(Equation S7)}$$

$$I(q) = scale * q^{-power} + background \quad \text{(Equation S8)}$$

The radius of gyration, R_g, for cylinders is provided in Equation S7. This model was combined in some cases with an additional power law model which contains parameters for scale and power (Equation S8). The scale is not directly the volume fraction, and these additional parameters were added to account for any corrections to the background subtraction. The combined model also contains parameters for scale and background which multiply or add to all scattering intensity by a number.

General Methods. Air and moisture-sensitive reactions were performed in a glovebox under nitrogen atmosphere or using a standard Schlenk vacuum line under nitrogen. Analytical thin-layer chromatography (TLC) was performed on aluminum-backed sheets coated with silica gel 60F254. Non-UV active compounds were detected on the plates by staining with 10% (w/v) phosphomolybdic acid in ethanol. Flash column chromatography was performed using a CombiFlash system with RediSep normal phase silica columns (Teledyne ISCO, silica gel 60, 230−400 mesh). HRMS(ESI) was performed with the Agilent LC-UV-TOF consisting of a 1260 UPLC, a UV-Vis diode-array detector (DAD) and a TOF mass analyzer. Chloroform-*d* ($CDCl_3$), methylene chloride-d_2 (CD_2Cl_2), and deuterium oxide (D_2O) were purchased from Cambridge Isotope Laboratories (Tewksbury, MA) and used for the collection of nuclear magnetic resonance (NMR) spectra. NMR spectra were recorded on a Bruker Ascend 700 spectrometer (^{1}H-700 MHz, ^{13}C-176 MHz), a Bruker 500 Avance spectrometer (^{1}H-500 MHz, ^{13}C-125 MHz), and a Bruker 400 Nanobay spectrometer (^{1}H-400 MHz, ^{13}C-100 MHz). Chemical shifts reported are in parts per million relative to the residual solvent peaks.

General preparation of poly(1′)$_{100}$ and poly(1)$_{100}$. Grubbs' third generation catalyst **4** was prepared as described by the literature.[2] D-Mannose (**1a′**), L-fucose (**1b′**), poly(**1a**)$_{100}$, poly(**1b**)$_{100}$, and acetylated precursors were prepared as described by the literature with matching spectra.[3,4]

General Preparation of poly(1′)$_{50}$. To a septum-sealed vial containing nitrogen and a stir bar, a solution of **4** (1.8 mM, 1 equiv) in dichloromethane (600 µL) was added and chilled to 0 °C for 10 min. A solution of **1a′** or **1b′** (90.2 mM, 50 equiv) in dichloromethane (400 µL) was then added to the vial and the reaction was initiated at 0 °C for 10 min. The reaction mixture was stirred at 25 °C for an additional 90 min. An excess of ethyl vinyl ether (0.1 mL) was used to terminate the reaction and left to stir for 30 min at 25 °C. Ethyl ether chilled to –20 °C was then used to precipitate the polymer, resulting in an off-white solid. After drying in vacuo to remove residual solvents, the polymer was analyzed by GPC and NMR to determine dispersity and purity.

poly(**1a′**)$_{50}$: (Yields: 48–61%) ^{1}H NMR (700 MHz, $CDCl_3$) δ 7.35–7.30 (m), 6.01 (br, s), 5.48–5.13 (m), 4.80 (br, s), 4.25 (br, s), 4.08 (br, s), 3.94 (br, s), 3.75 (br, s), 3.69–3.15 (m), 3.01 (br, s), 2.67 (br, s), 2.32 (br, s), 2.14 (br, s), 2.08 (br, s), 2.04 (br, s), 1.99 (br, s), 1.94–1.87 (m), 1.82 (br, s), 1.14 (br, s). ^{13}C NMR (176 MHz, $CDCl_3$) δ 174.87, 170.77, 170.16, 169.79, 133.87, 131.84, 97.86, 97.64, 69.41, 69.22, 68.75, 67.50, 66.12, 62.52, 52.18, 48.27, 43.69, 42.15, 39.05, 36.95, 21.00, 20.89, 20.85.

poly(**1b′**)$_{50}$: (Yields: 60–66%) ^{1}H NMR (700 MHz, $CDCl_3$) δ 7.36–7.31 (m), 5.91 (br, s), 5.47–5.08 (m), 5.02 (br, s), 4.12 (br, s), 3.72 (br, s), 3.51 (br, s), 3.35 (br, s), 3.03 (br, s), 2.69 (br, s), 2.25 (br,

s), 2.16 (br, s), 2.06 (br, s), 1.98 (br, s), 1.96–1.86 (m), 1.67 (br, s), 1.12 (br, s). ^{13}C NMR (176 MHz, $CDCl_3$) δ 174.34, 170.76, 170.31, 133.71, 131.47, 96.57, 71.17, 68.25, 68.08, 67.85, 64.74, 52.58, 48.25, 43.69, 42.16, 39.22, 37.02, 21.04, 20.88, 20.81, 16.07.

General Preparation of poly(1)$_{50}$. To a reaction vial containing poly(**1′**)$_{50}$ (50 mg), K_2CO_3 was added in excess. 4.0 mL of an anhydrous mixture of MeOH:THF (2:1 v/v) was added to the vial and the reaction was left to stir at 25 °C for 90 min. The reaction mixture was concentrated in vacuo, neutralized using 5.0 mL of 1 N HCl in H_2O:THF (1:1 v/v), and left to stir for 90 min. The reaction mixture was transferred to a prewetted cellulose ester Spectra/Por® Float-A-Lyzer® G2 dialysis device (MWCO 3.5–5kD, 5 mL) and dialyzed against DI water for at least 3 d. The mixture was then lyophilized for 2 d to afford an off-white solid.

poly(**1a**)$_{50}$: (Yields: 67–88%) ^{1}H NMR (400 MHz, D_2O) δ 7.48–7.24 (m), 5.54–5.21 (m), 4.91–4.84 (m), 3.94 (br, s), 3.90–3.51 (m), 3.41 (br, s), 3.02 (br, s), 2.82 – 2.59 (m), 2.52 (br, s), 2.02 (br, s), 1.68 (br, s), 1.22 (br, s).

poly(**1b**)$_{50}$: (Yields: 80–99%) ^{1}H NMR (400 MHz, D_2O) δ 7.48–7.28 (m), 5.56–5.19 (m), 4.87 (br, s), 3.99 (br, s), 3.90–3.67 (m), 3.62–3.27 (m), 3.05 (br, s), 2.71 (br, s), 2.51 (br, s), 2.25–1.78 (m), 1.66 (br, s), 1.22 (br, s).

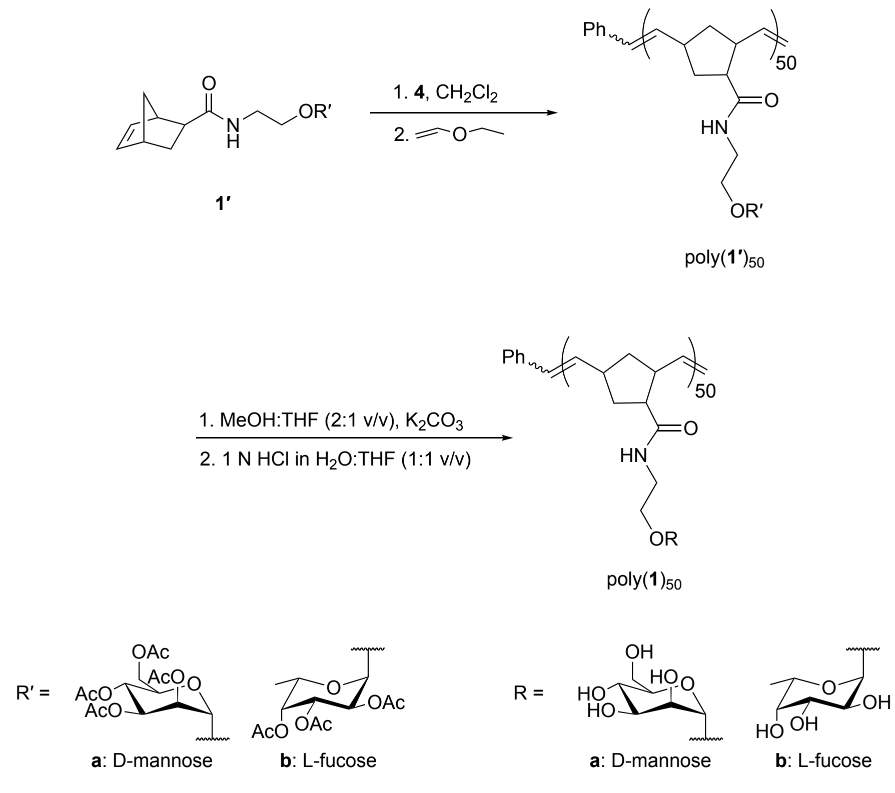

Scheme S1. Synthesis of poly(**1′**)$_{50}$ and poly(**1**)$_{50}$

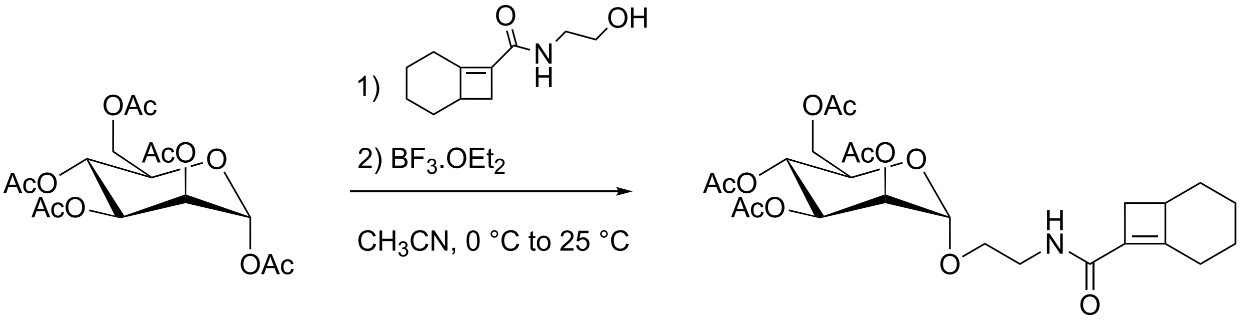

Scheme S2. Synthesis of 2,3,4,6-tetra-O-acetyl-α-D-mannopyranosyl bicyclo[4.2.0]oct-6-ene-7-carboxamide

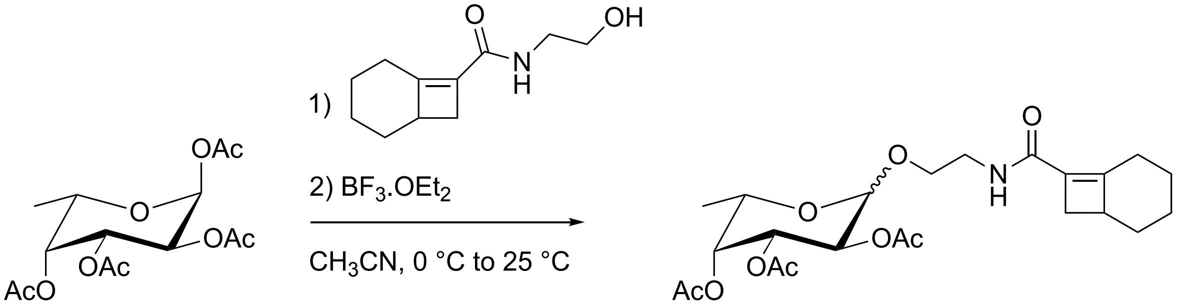

Scheme S3. Synthesis of 2,3,4-tri-O-acetyl-L-fucopyranosyl bicyclo[4.2.0]oct-6-ene-7-carboxamide

Table S1. Protein conjugate concentrations and degree of labeling for SBTI-Alexa 488 conjugates prepared.

Conjugate Date	[Protein] (μM)[a]	Moles of dye: mole of protein[a]
2/27/20	15.3	1.45
8/27/20	21.0	1.07
5/3/21	15.4	0.85
7/13/21	25.4	1.18
2/1/22	21.3	1.03
5/4/22	14.6	1.88
12/8/22	18.4	2.70
3/27/23	18.8	3.13
**Average**	18.8	1.66

[a]Conjugate concentration and degree of labeling were calculated using Equation S1 and Equation S2, respectively.

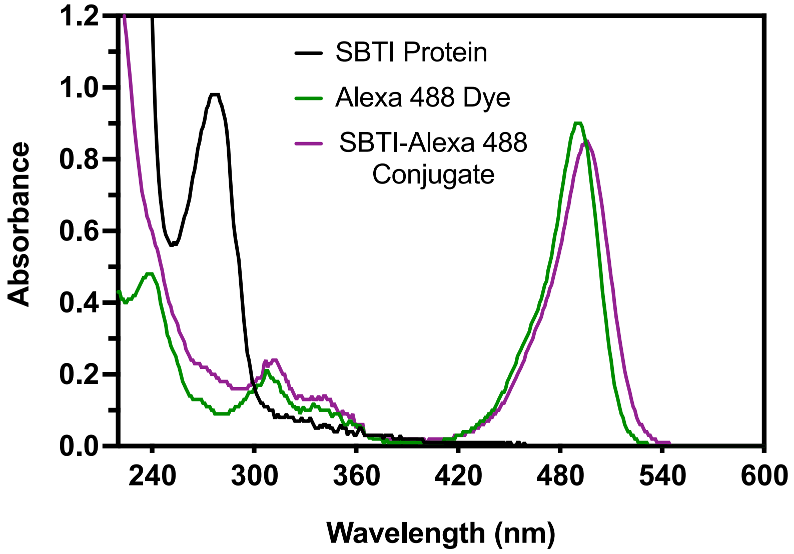

Figure S1. Spectral overlay of the UV-vis traces of SBTI protein (black trace), Alexa Fluor™ 488 dye (green trace), and SBTI-Alexa 488 conjugate (purple trace) between 240 and 600 nm.

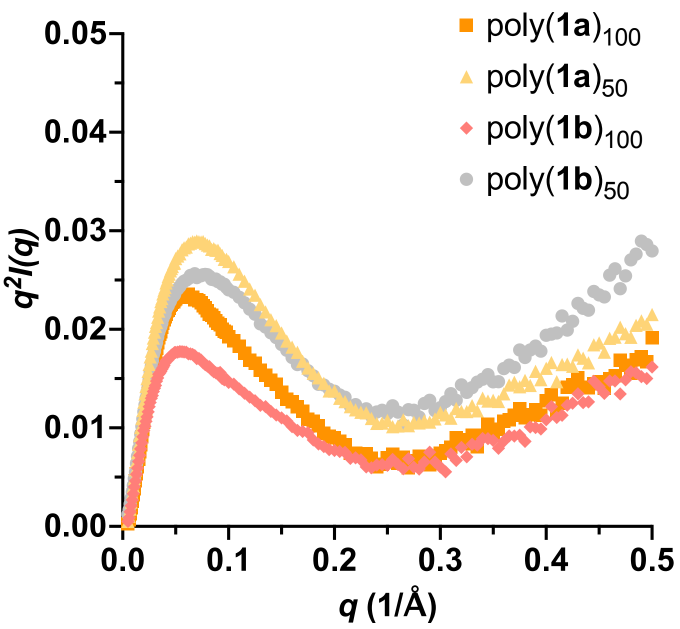

Figure S2. Kratky plots of poly(**1**)$_{100}$ and poly(**1**)$_{50}$

The Kratky plot shows q vs. $q^2 * I(q)$. This can give insight into the arrangement or folding of scatterers in solution. The Kratky plots for poly(**1**)$_{100}$ and poly(**1**)$_{50}$ show a broad peak at low-q, followed by a steady increase at higher q. This is usually correlated to partially unfolded proteins or slightly swollen polymer chains.[6] This is consistent with the flexible cylinder model used to fit the data as it suggests that the polymers may be coiling or folding due to more favorable interactions between polymer chains rather than the solvent.

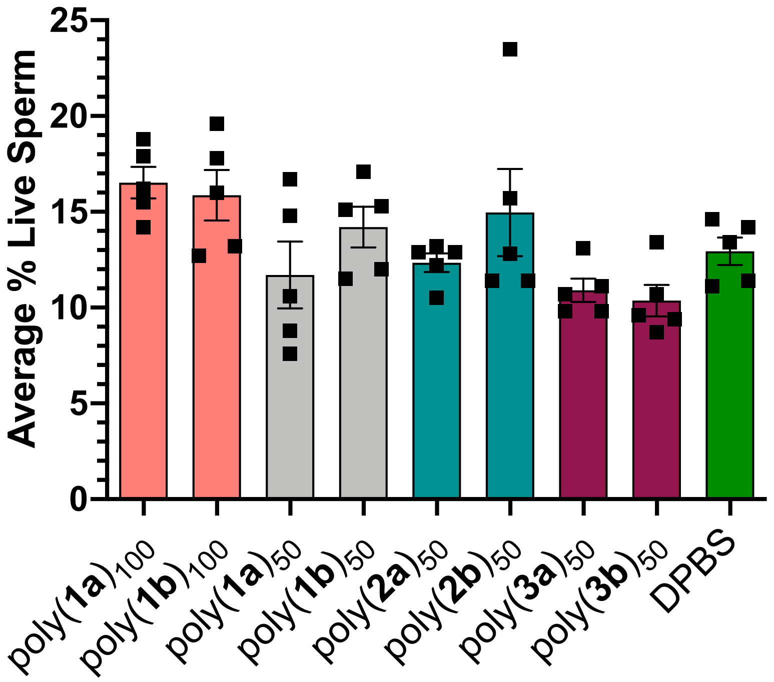

Figure S3. Comparison of the average percent of live mouse sperm after treatment with glycopolymers or the negative control (DPBS). The average percentage of live sperm treated with DPBS was 13%. Data represents mean ± standard error of the mean of the cell viability at each of the five polymer concentrations. One-way ANOVA was used to compare the average cell viability of glycopolymers to the average cell viability of the negative control where $*p < 0.05$, $**p < 0.01$, $***p < 0.001$, and $****p < 0.0001$. There was no statistically significant difference between the cell viability of any of the samples treated with glycopolymers or the negative control.

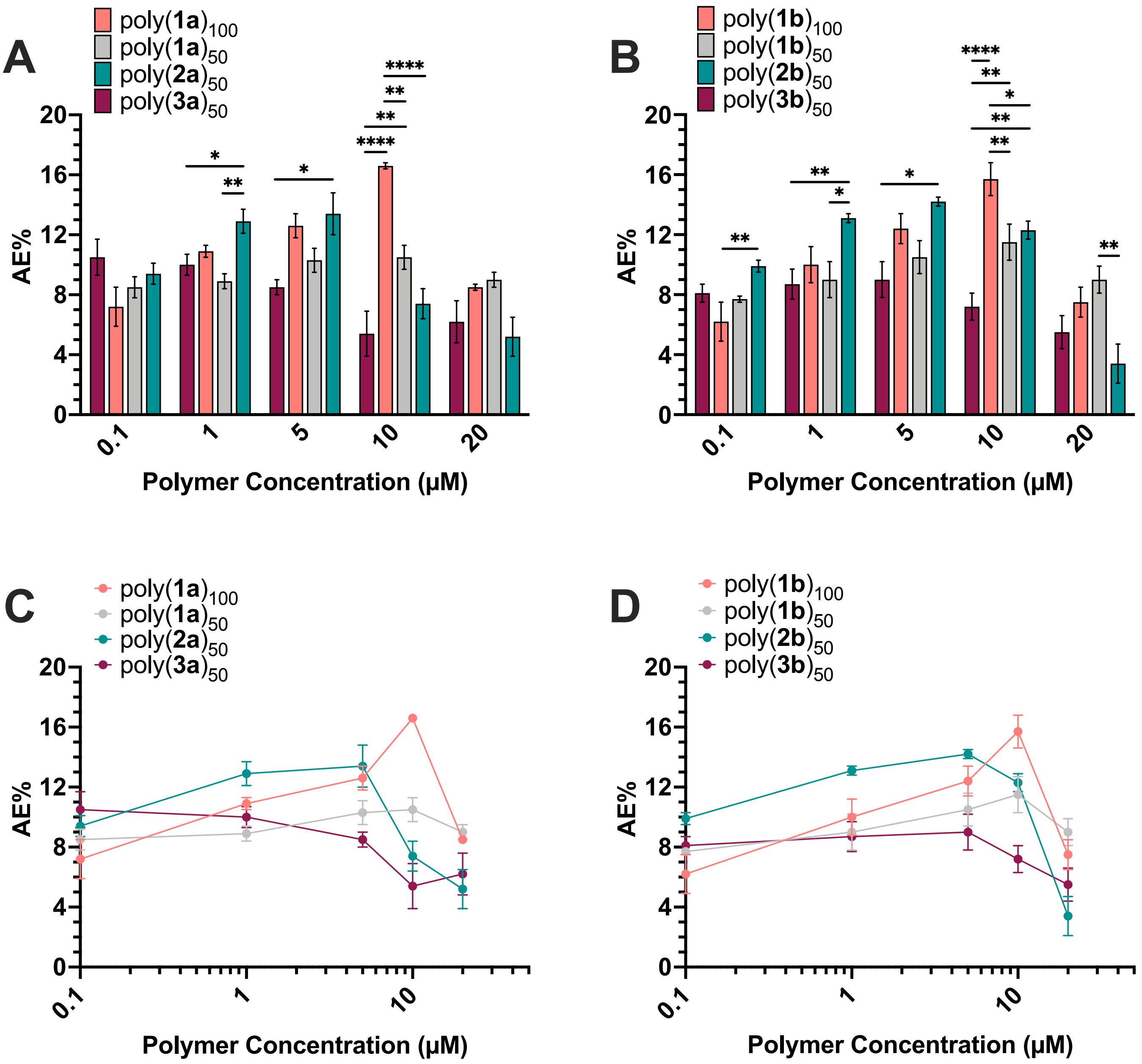

Figure S4. Comparison of AE induction in mouse sperm by (**A**) mannose or (**B**) fucose glycopolymers. Activation curves of (**C**) mannose and (**D**) fucose glycopolymers are shown on a $\log_{10}$ scale. The average AE% for mouse sperm treated with DPBS (negative control) was 7.2%. Data represents mean ± standard error of the mean of at least three independent experiments testing two batches of each polymer. One-way ANOVA was used to compare AE% where $*p < 0.05$, $**p < 0.01$, $***p < 0.001$, and $****p < 0.0001$.

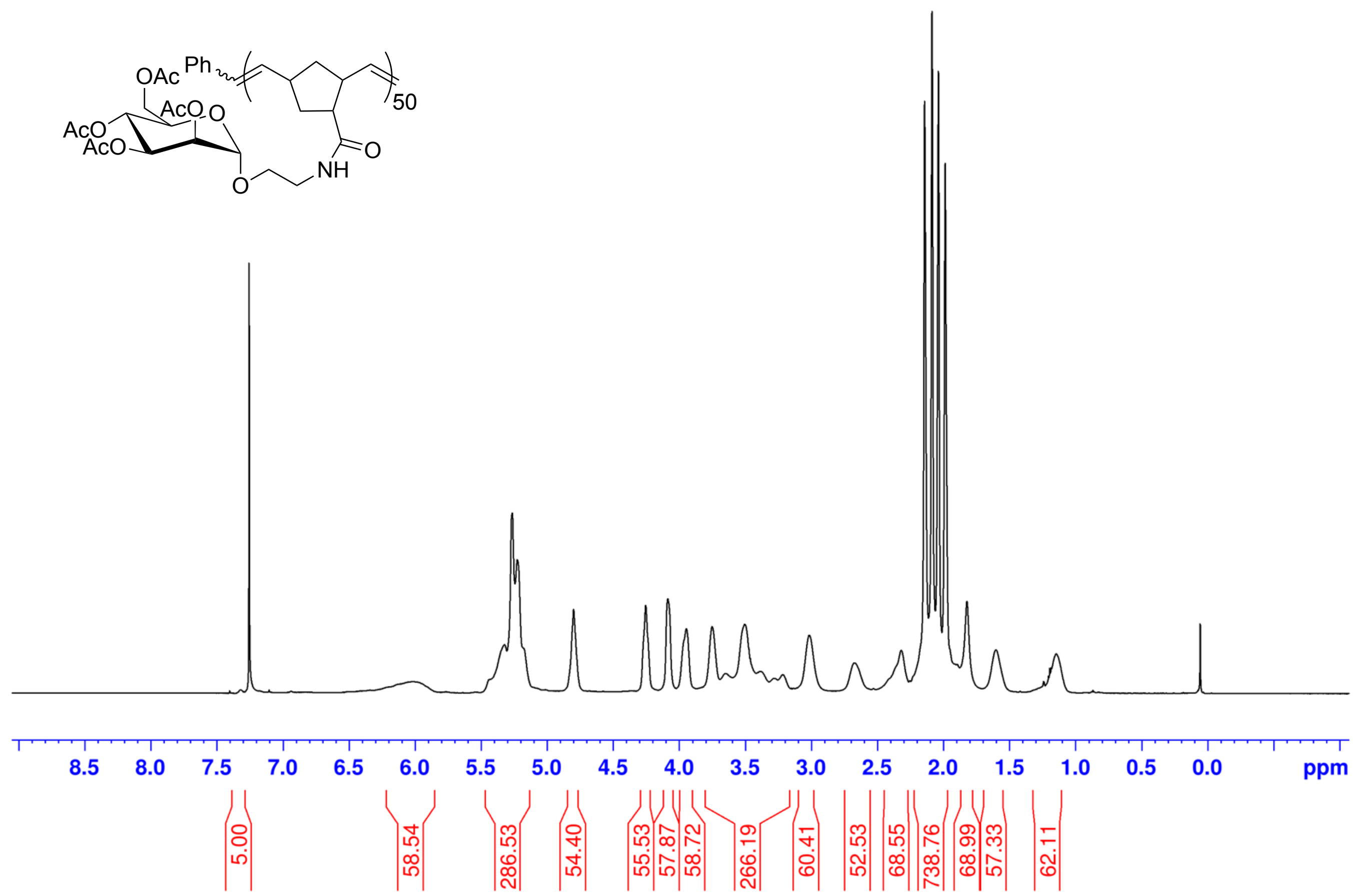

Figure S5. ^{1}H NMR (700 MHz, $CDCl_3$) spectrum of poly(**1a′**)$_{50}$

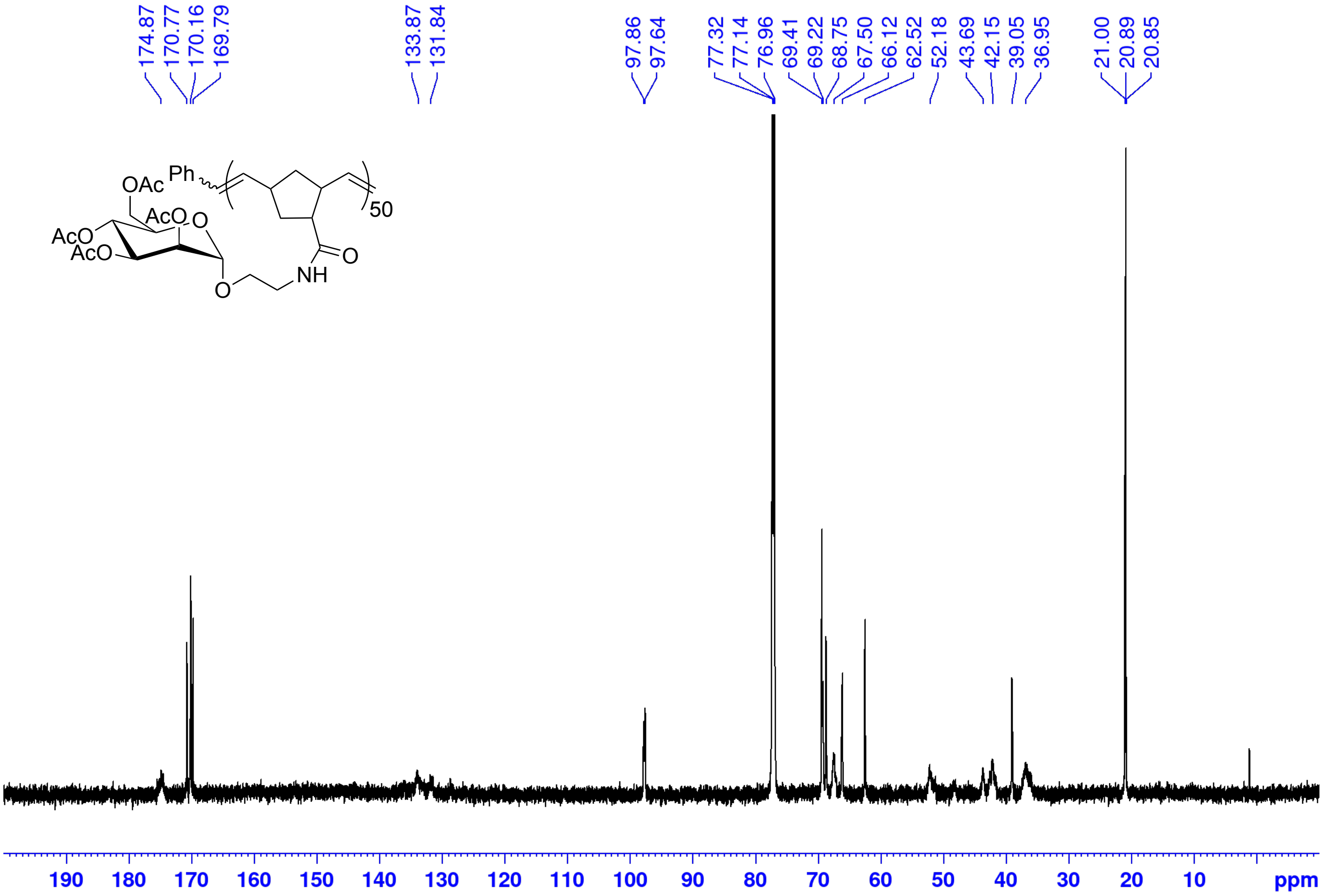

Figure S6. ^{13}C NMR (176 MHz, $CDCl_3$) spectrum of poly(**1a′**)$_{50}$

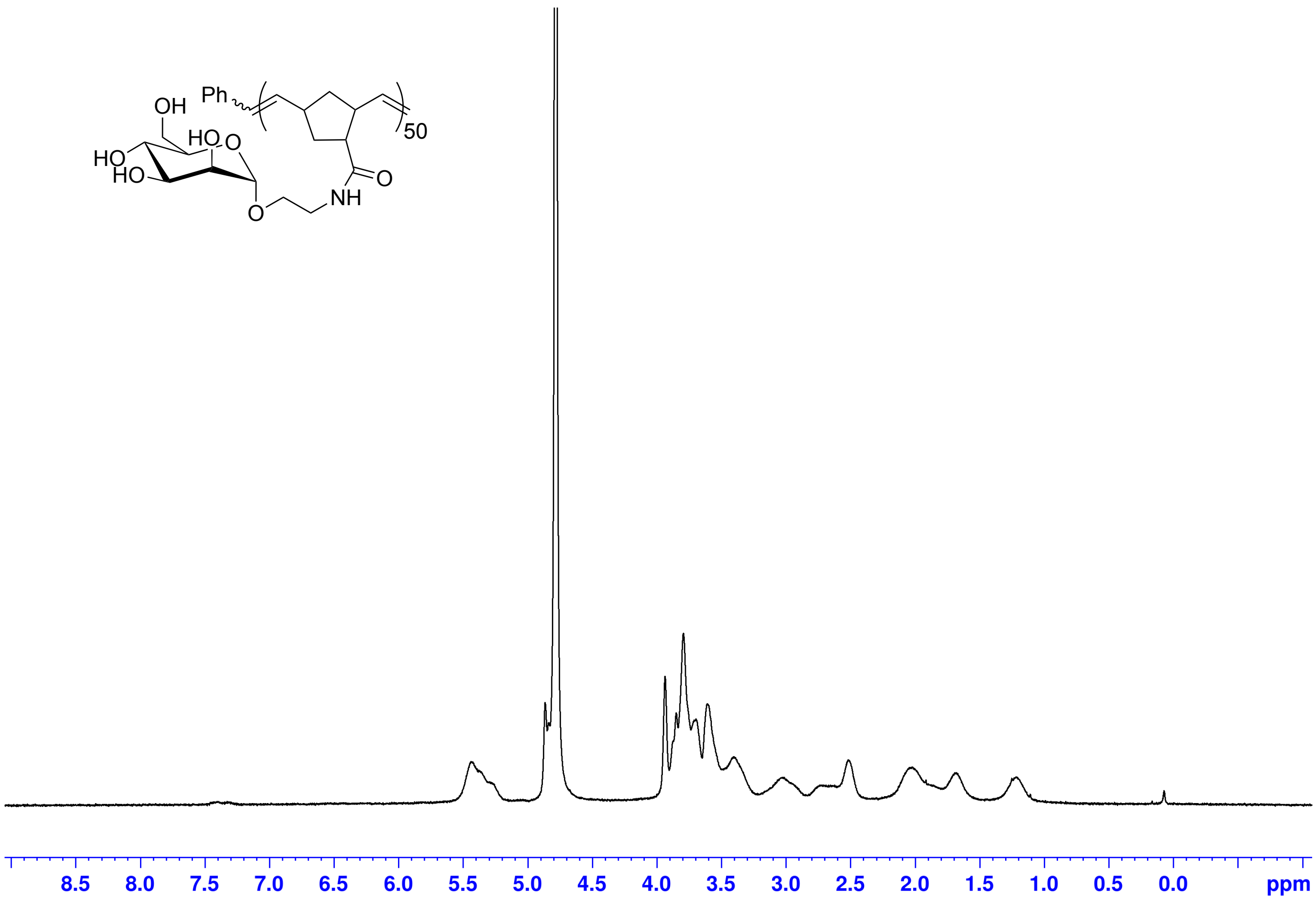

Figure S7. ^{1}H NMR (400 MHz, D_2O) spectrum of poly(**1a**)$_{50}$

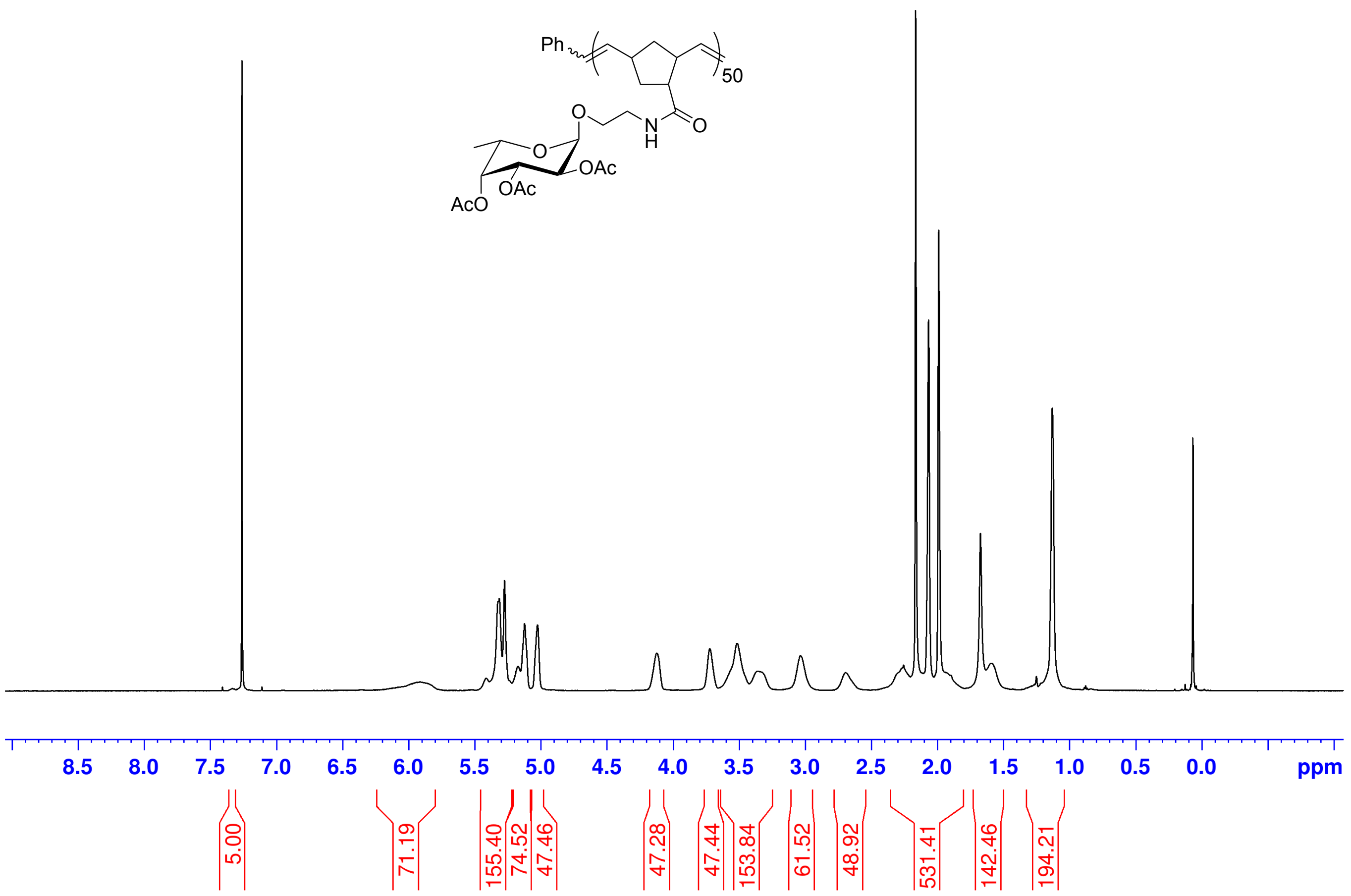

Figure S8. ^{1}H NMR (700 MHz, $CDCl_3$) spectrum of poly(**1b′**)$_{50}$

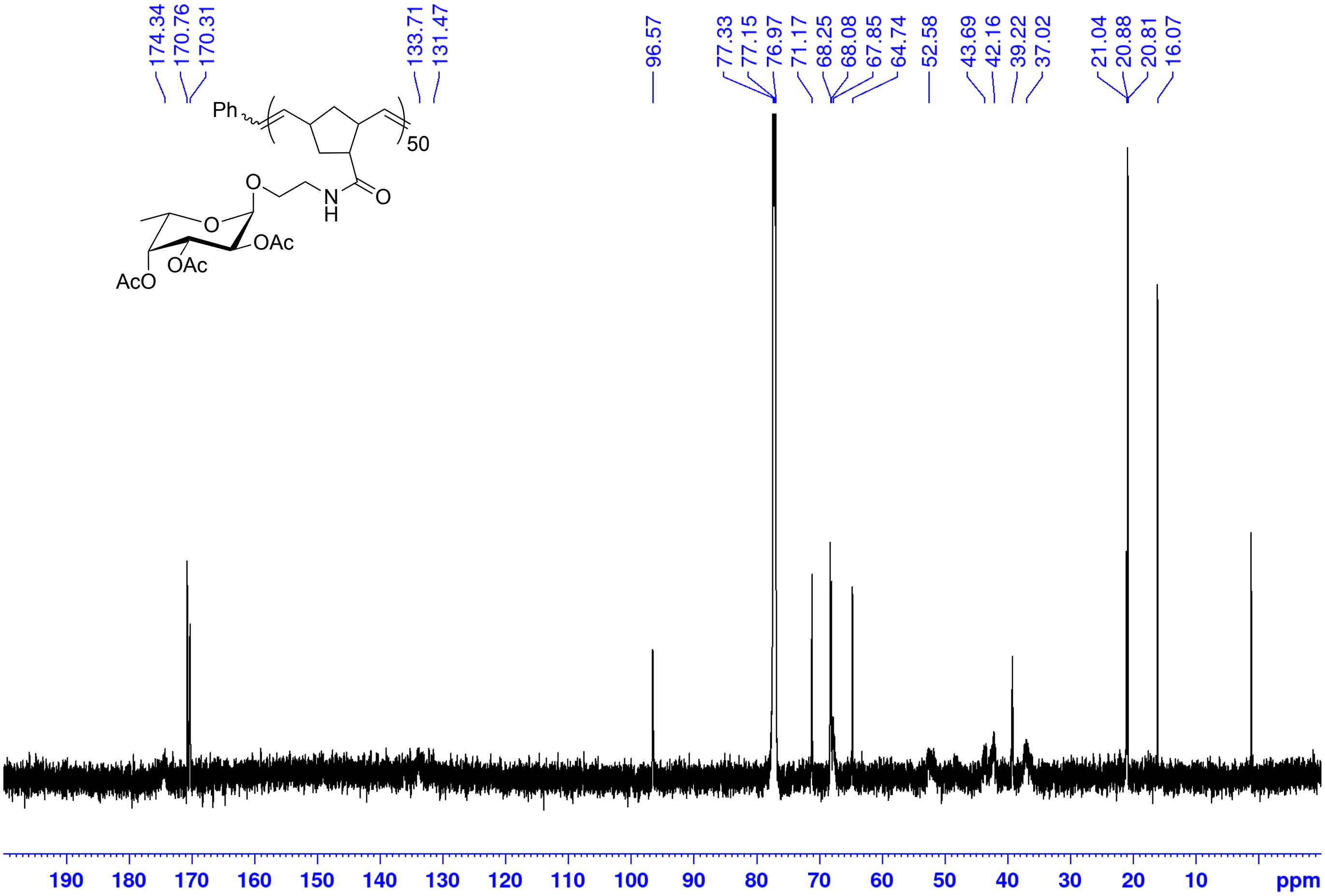

Figure S9. ^{13}C NMR (176 MHz, $CDCl_3$) spectrum of poly(**1b′**)$_{50}$

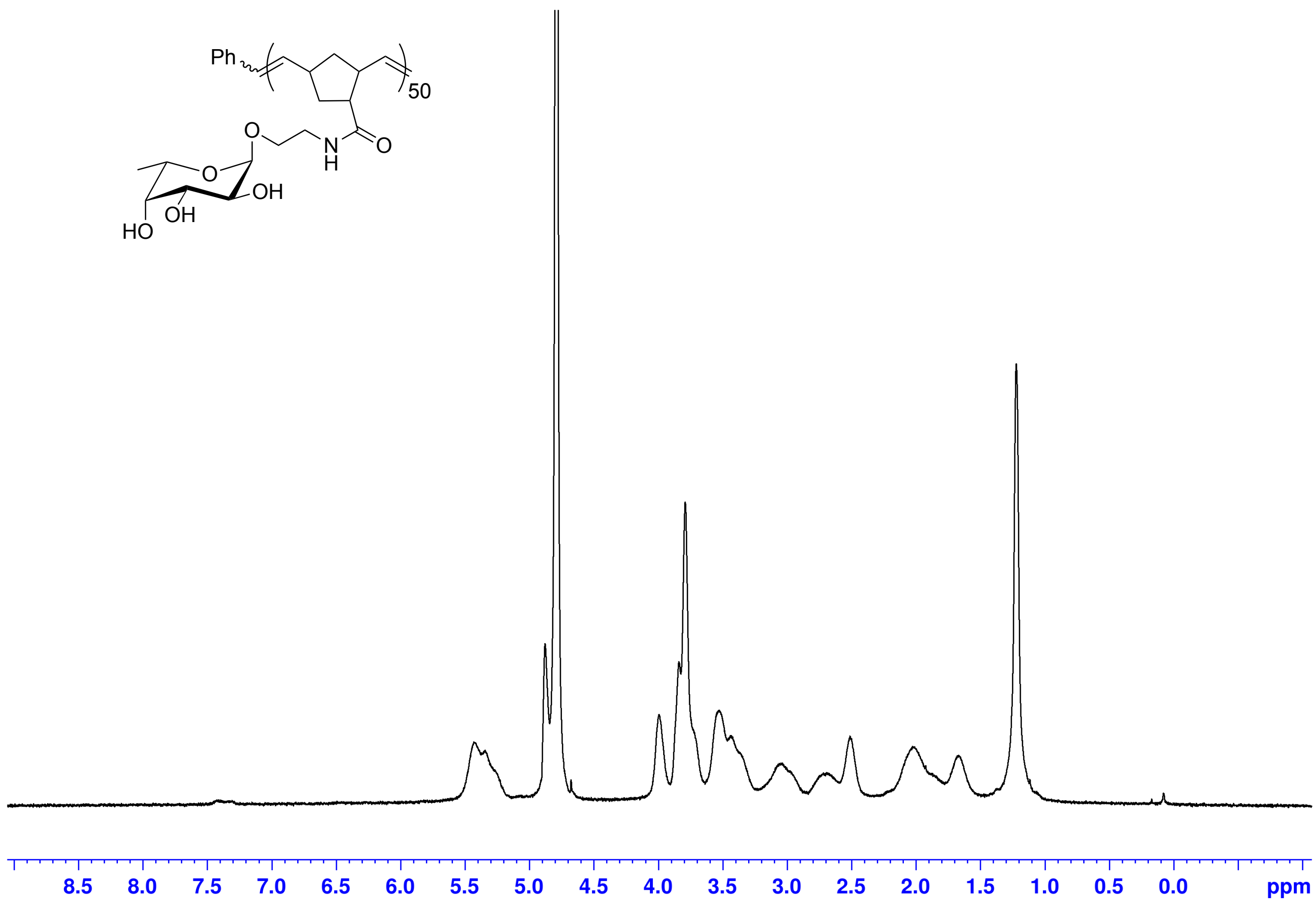

Figure S10. ^{1}H NMR (400 MHz, D_2O) spectrum of poly(**1b**)$_{50}$

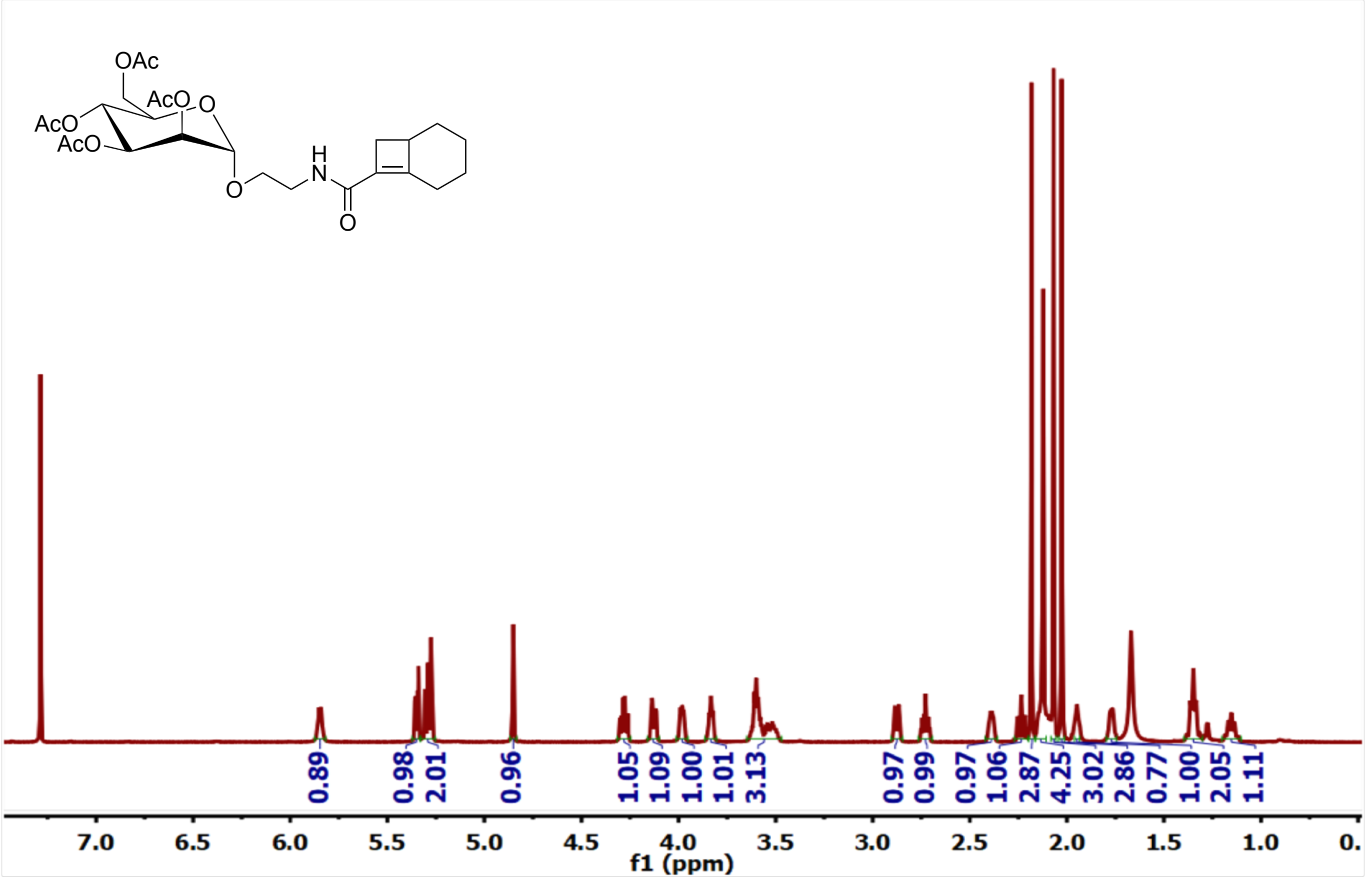

Figure S11. ^{1}H NMR (700 MHz, $CDCl_3$) spectrum of 2,3,4,6-tetra-O-acetyl-α-D-mannopyranosyl bicyclo[4.2.0]oct-6-ene-7-carboxamide

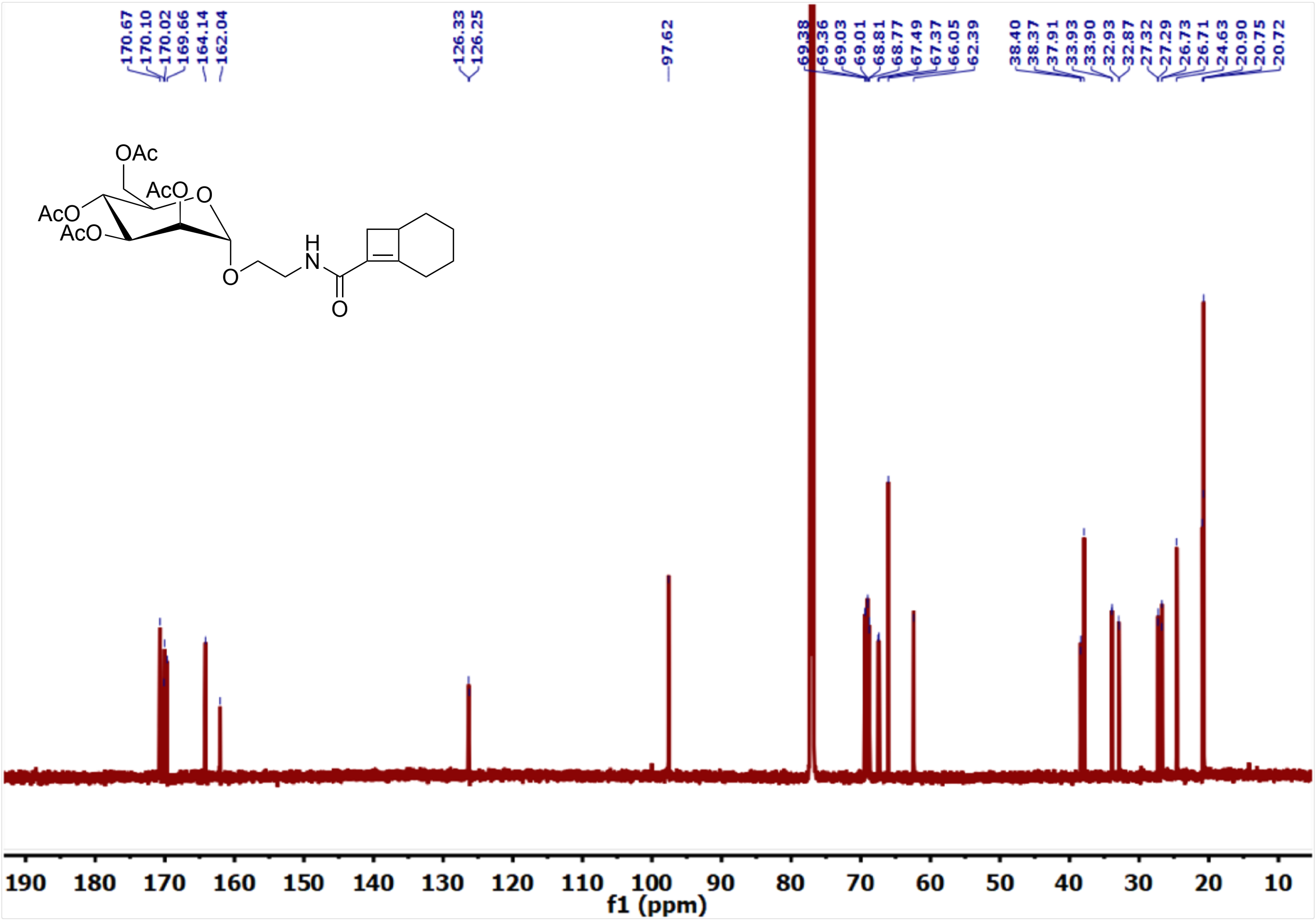

Figure S12. ^{13}C NMR (176 MHz, $CDCl_3$) spectrum of 2,3,4,6-tetra-O-acetyl-α-D-mannopyranosyl bicyclo[4.2.0]oct-6-ene-7-carboxamide

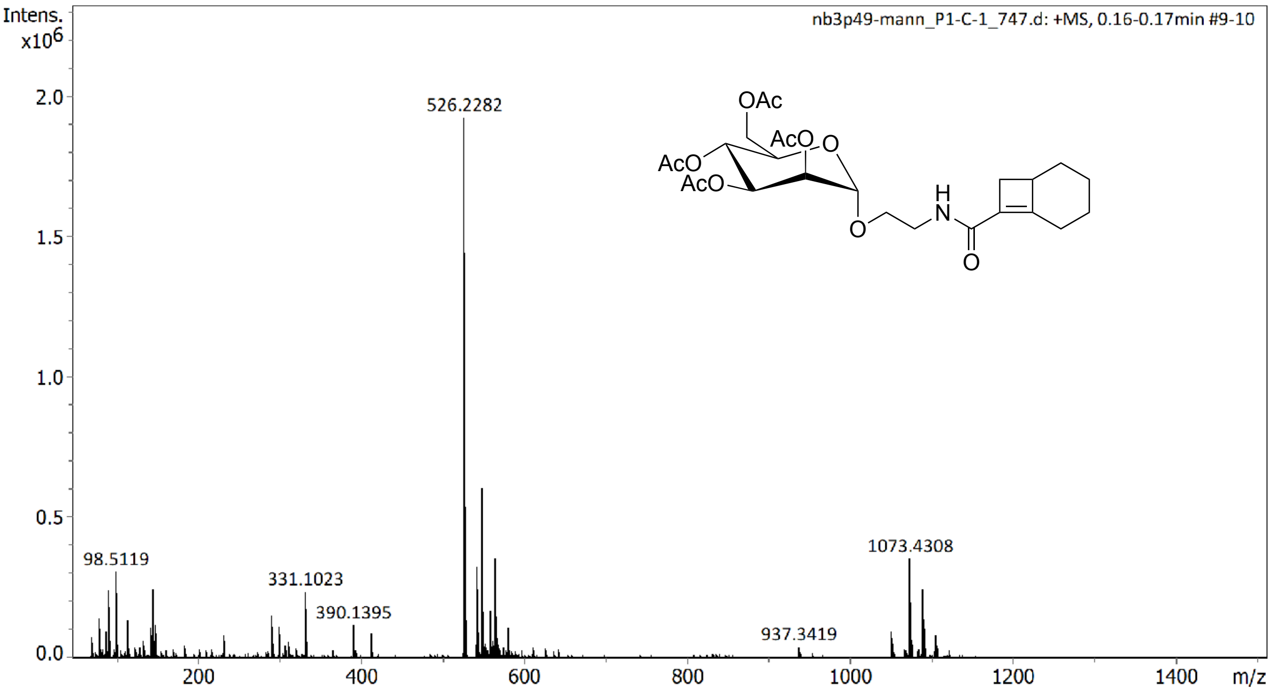

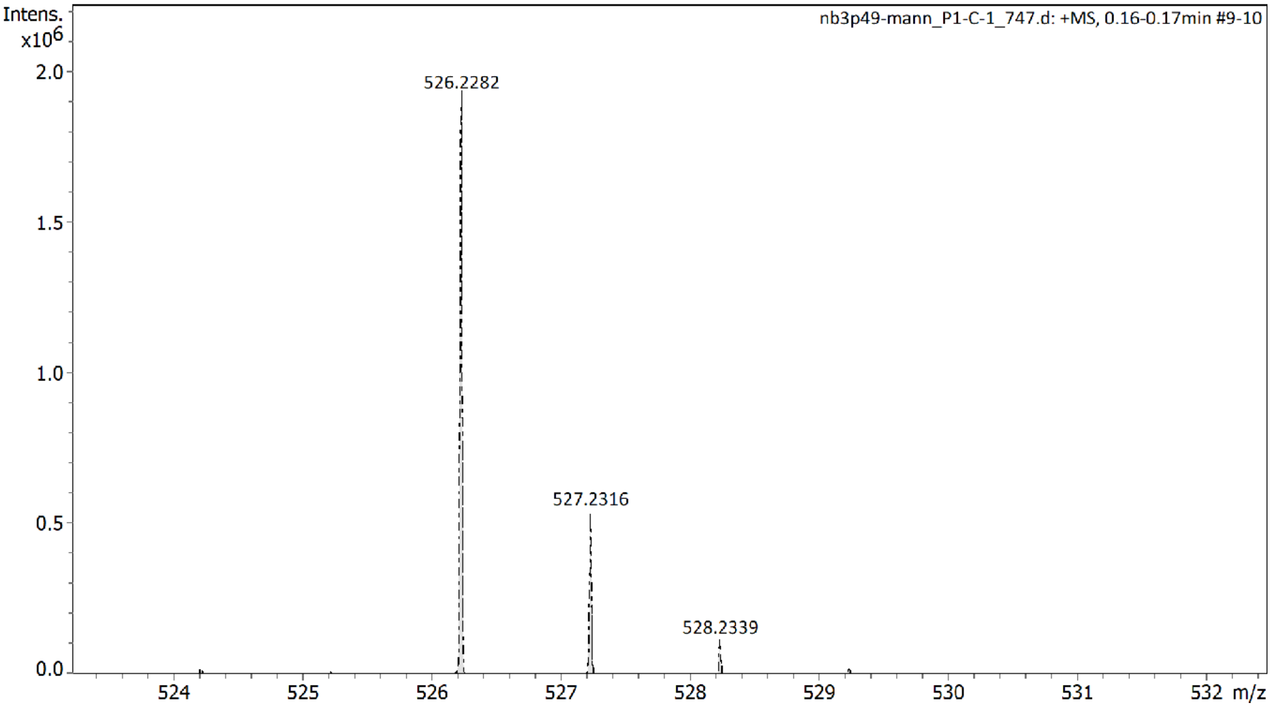

Figure S13. HRMS of 2,3,4,6-tetra-O-acetyl-α-D-mannopyranosyl bicyclo[4.2.0]oct-6-ene-7-carboxamide

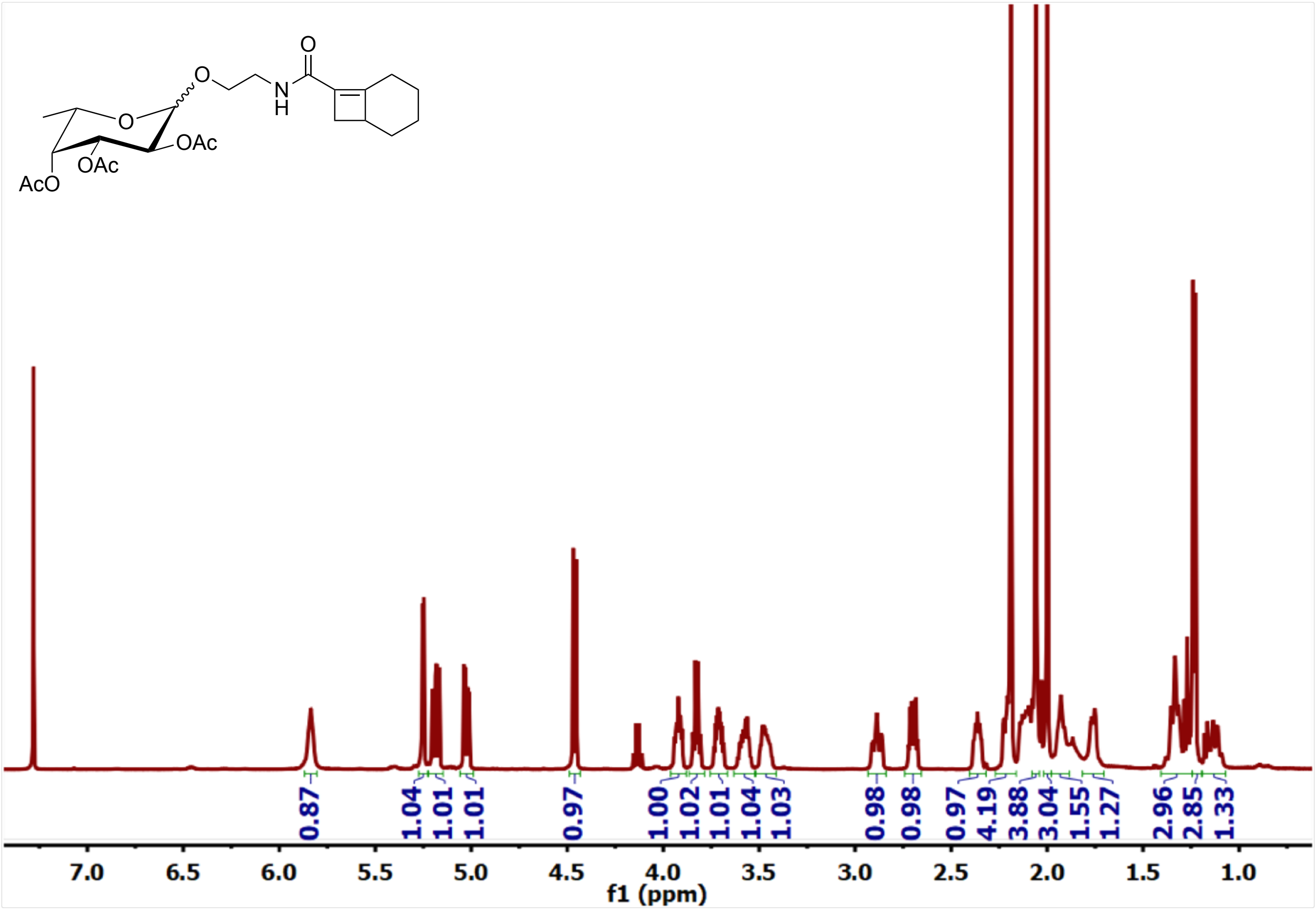

Figure S14. ^{1}H NMR (700 MHz, $CDCl_3$) spectrum of 2,3,4-tri-O-acetyl-L-fucopyranosyl bicyclo[4.2.0]oct-6-ene-7-carboxamide

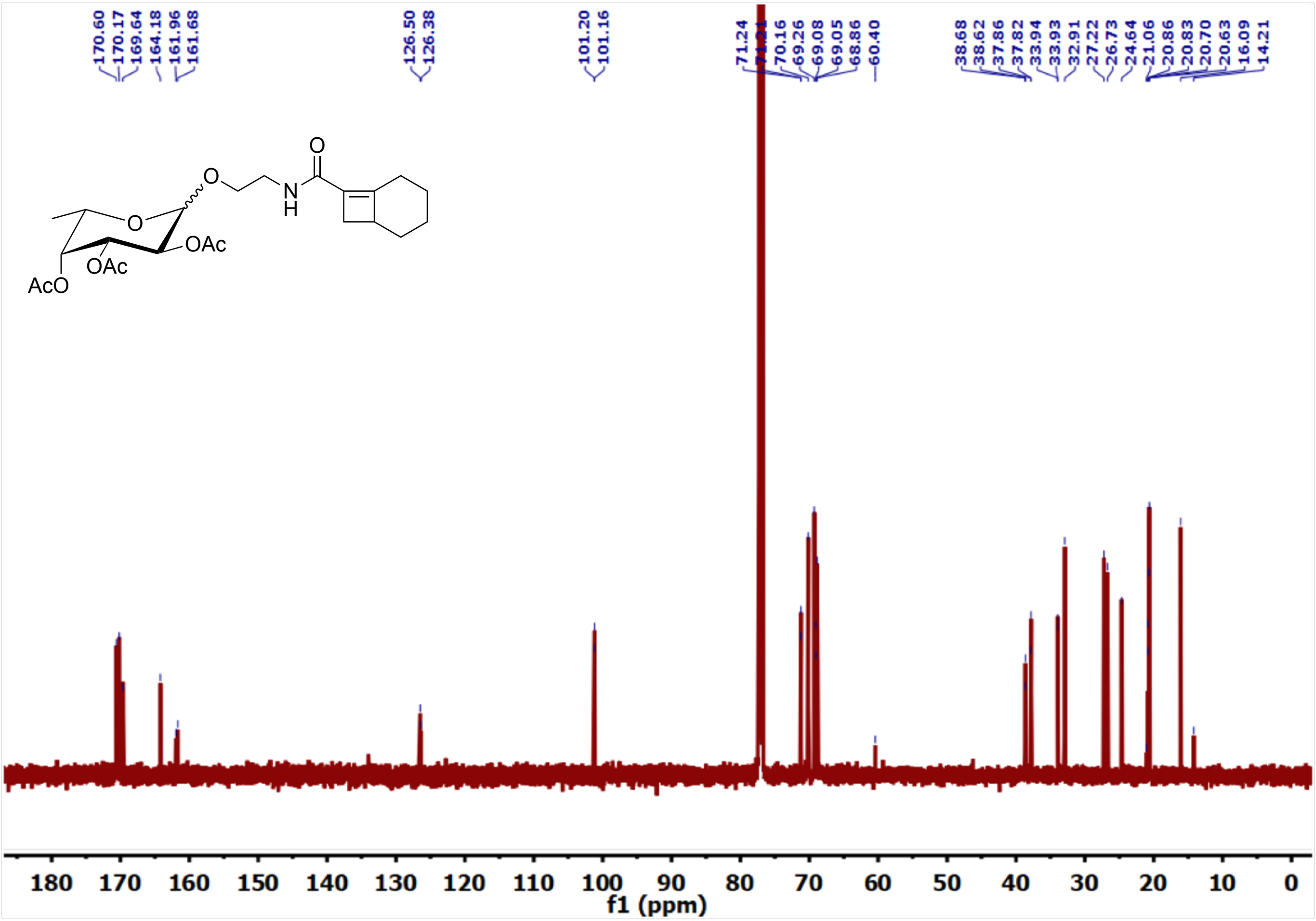

Figure S15. ^{13}C NMR (176 MHz, $CDCl_3$) spectrum of 2,3,4-tri-O-acetyl-L-fucopyranosyl bicyclo[4.2.0]oct-6-ene-7-carboxamide

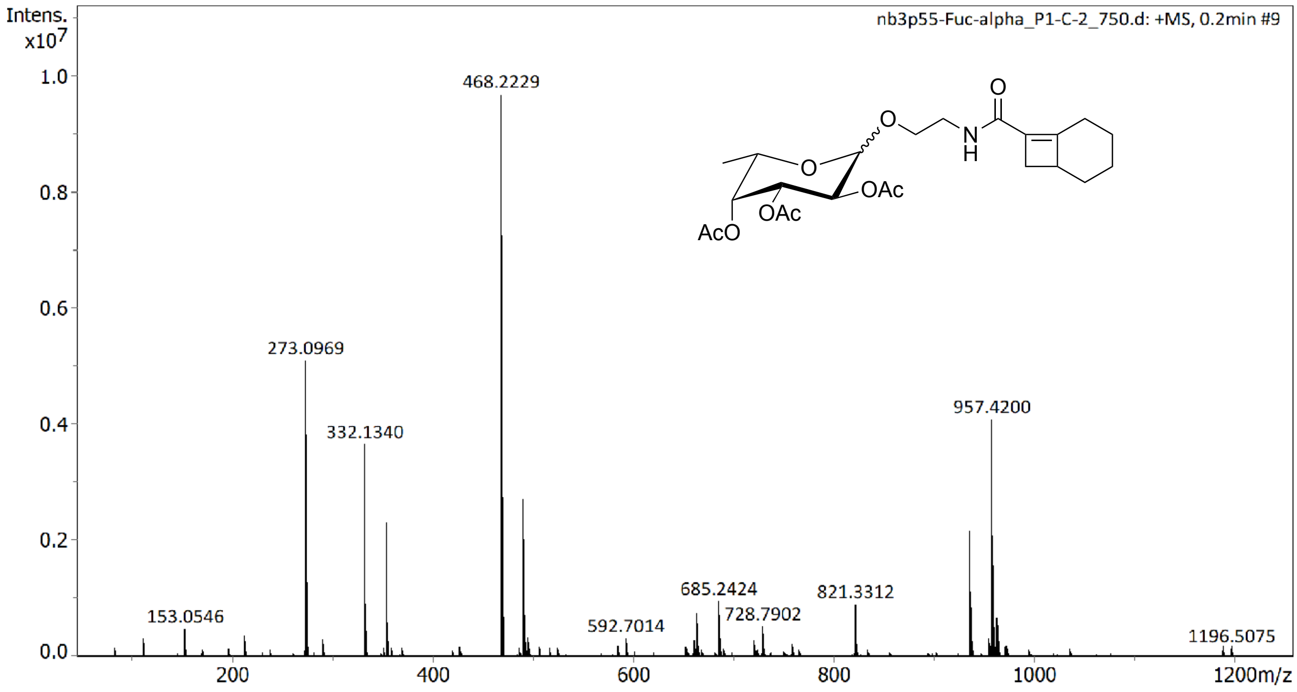

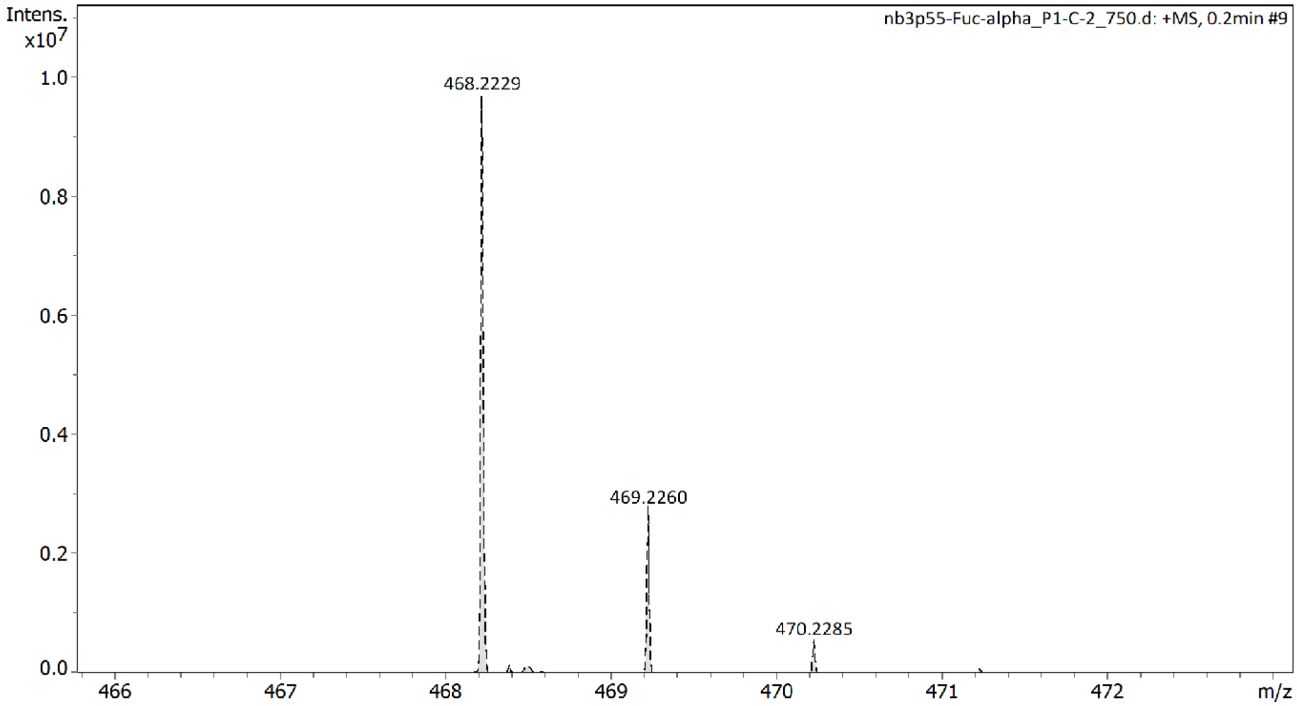

Figure S16. HRMS of 2,3,4-tri-O-acetyl-L-fucopyranosyl bicyclo[4.2.0]oct-6-ene-7-carboxamide

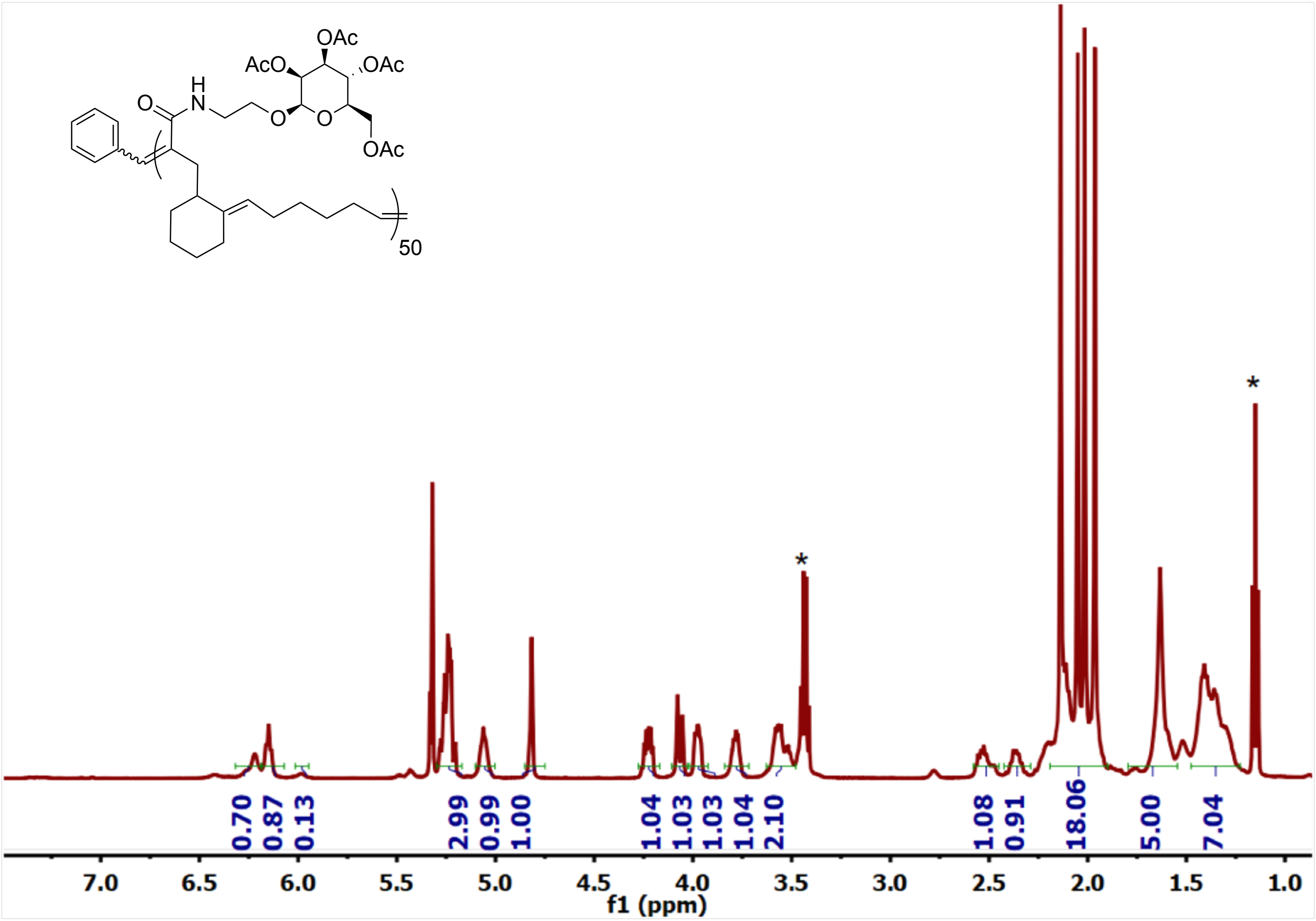

Figure S17. ^{1}H NMR (500 MHz, CD_2Cl_2) spectrum of poly(**2a′**)$_{50}$

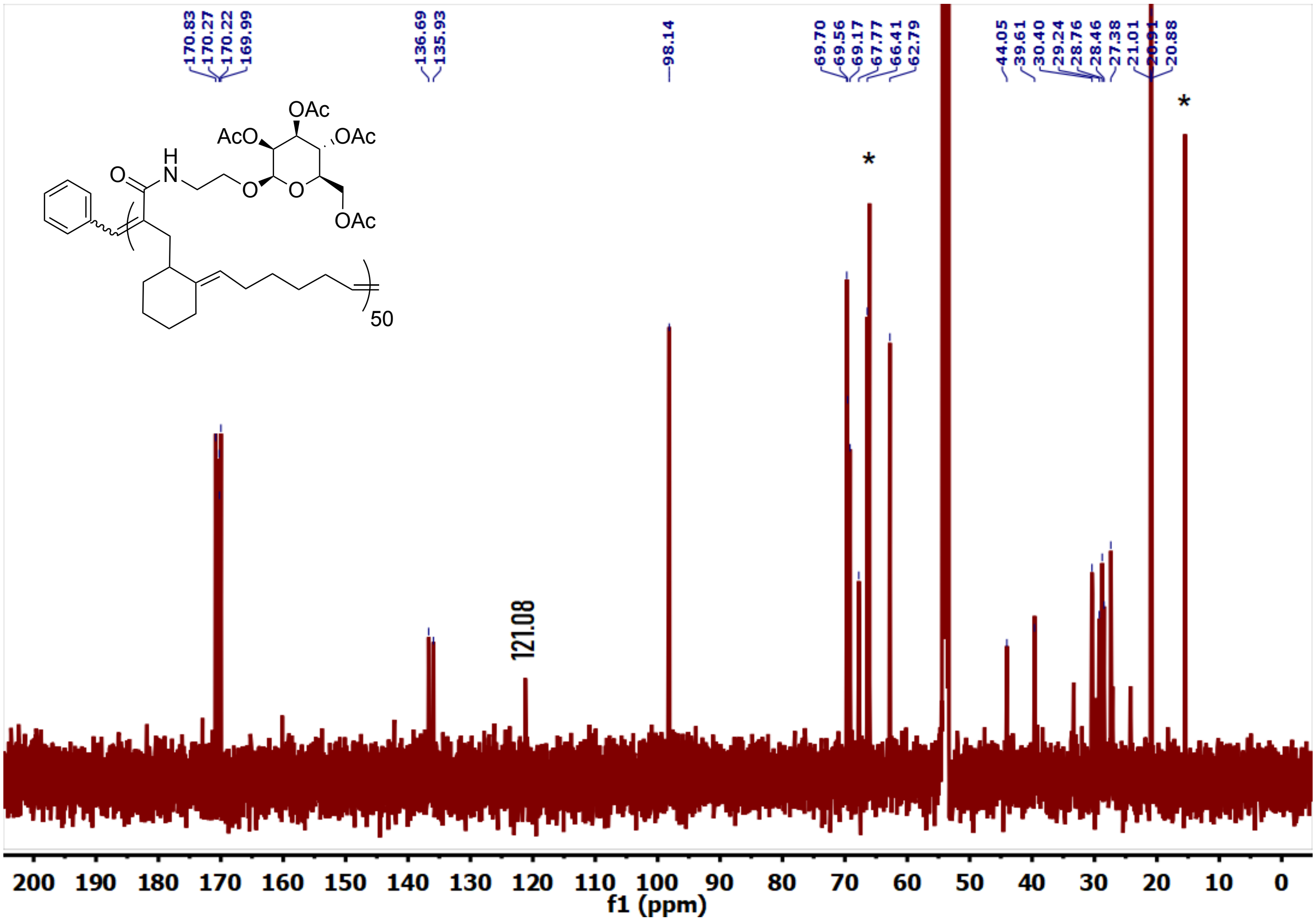

Figure S18. ^{13}C NMR (125 MHz, CD_2Cl_2) spectrum of poly(**2a′**)$_{50}$

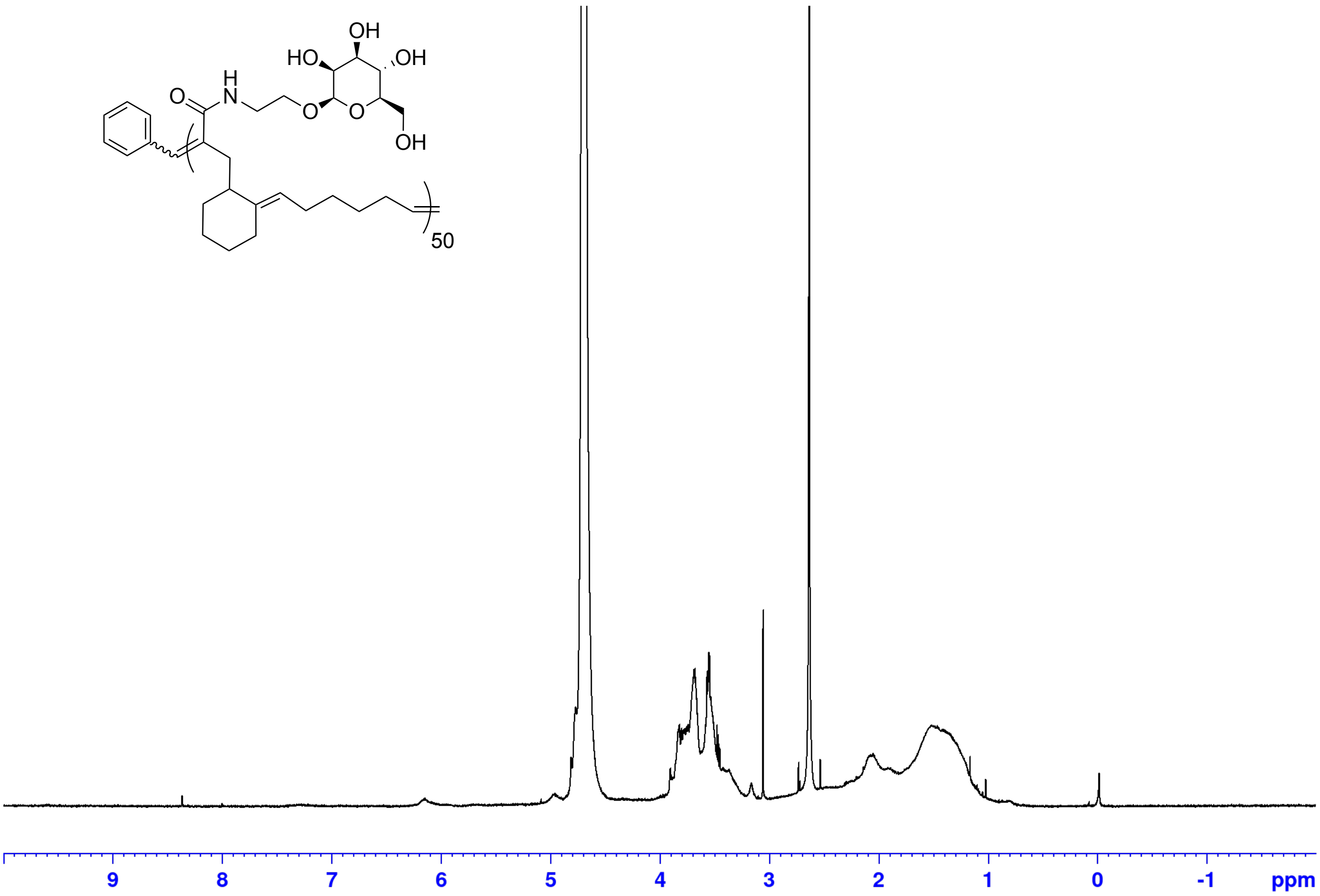

Figure S19. ^{1}H NMR (700 MHz, D_2O) spectrum of poly(**2a**)$_{50}$

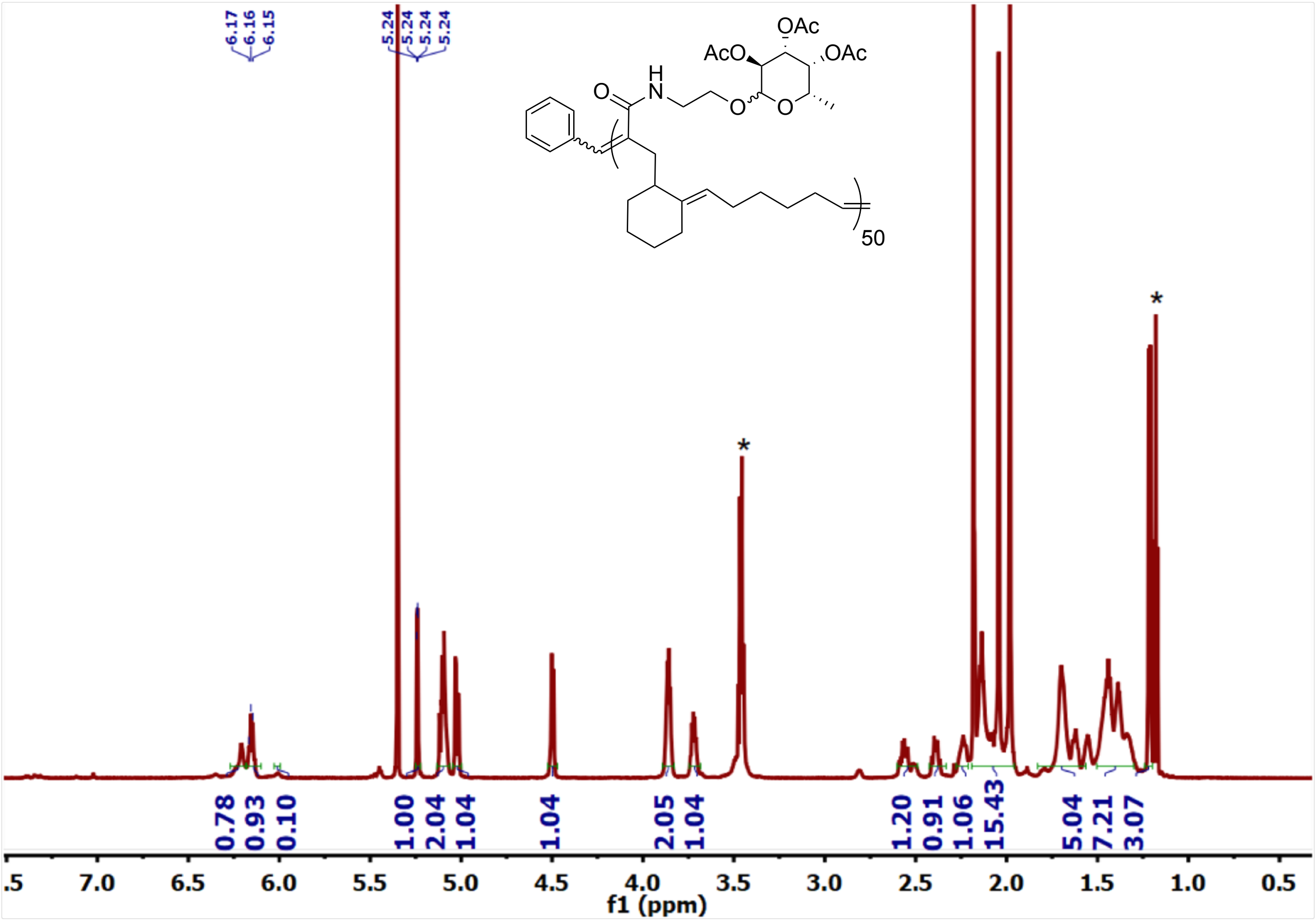

Figure S20. ^{1}H NMR (700 MHz, CD_2Cl_2) spectrum of poly(**2b′**)$_{50}$

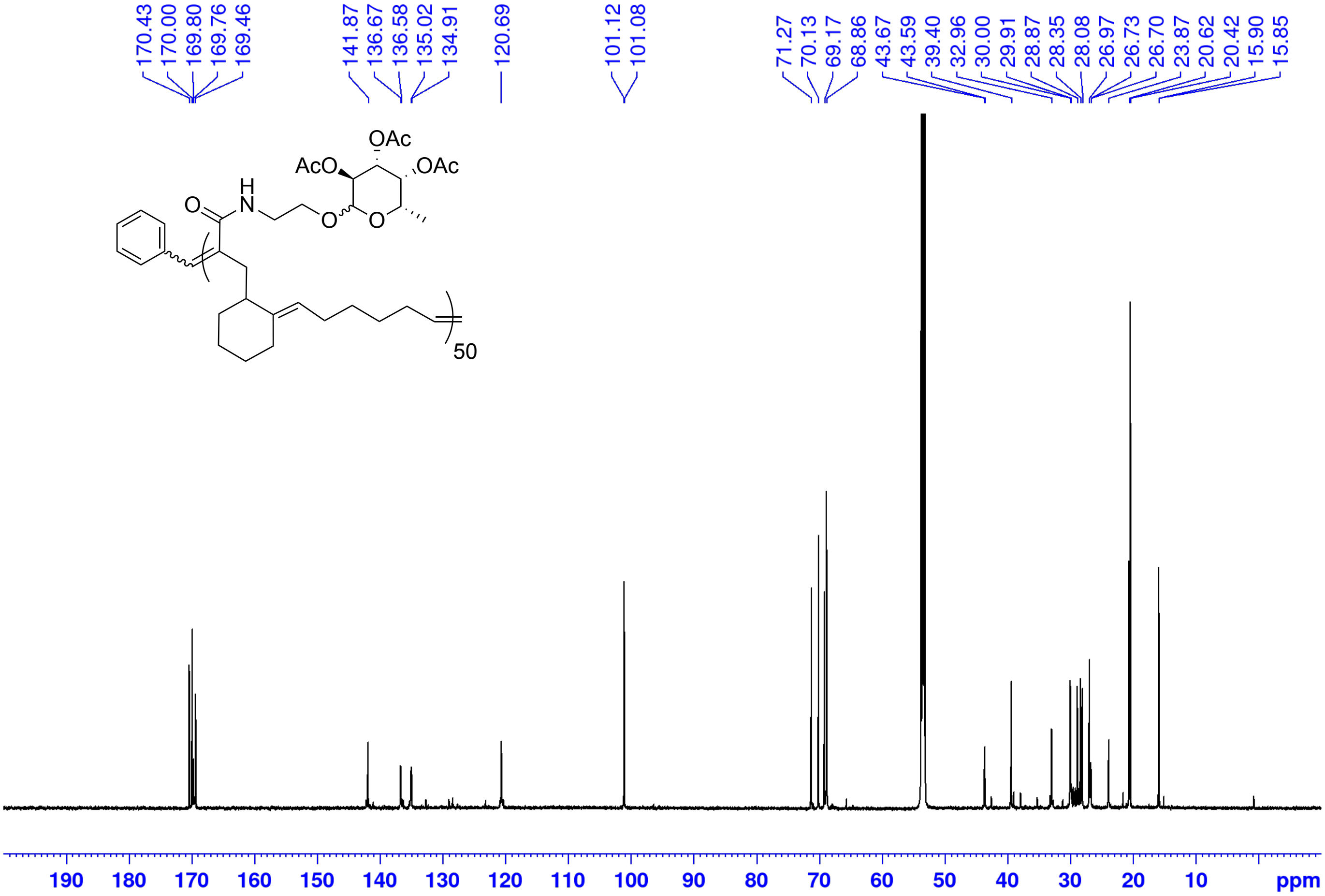

Figure S21. ^{13}C NMR (176 MHz, CD_2Cl_2) spectrum of poly(**2b′**)$_{50}$

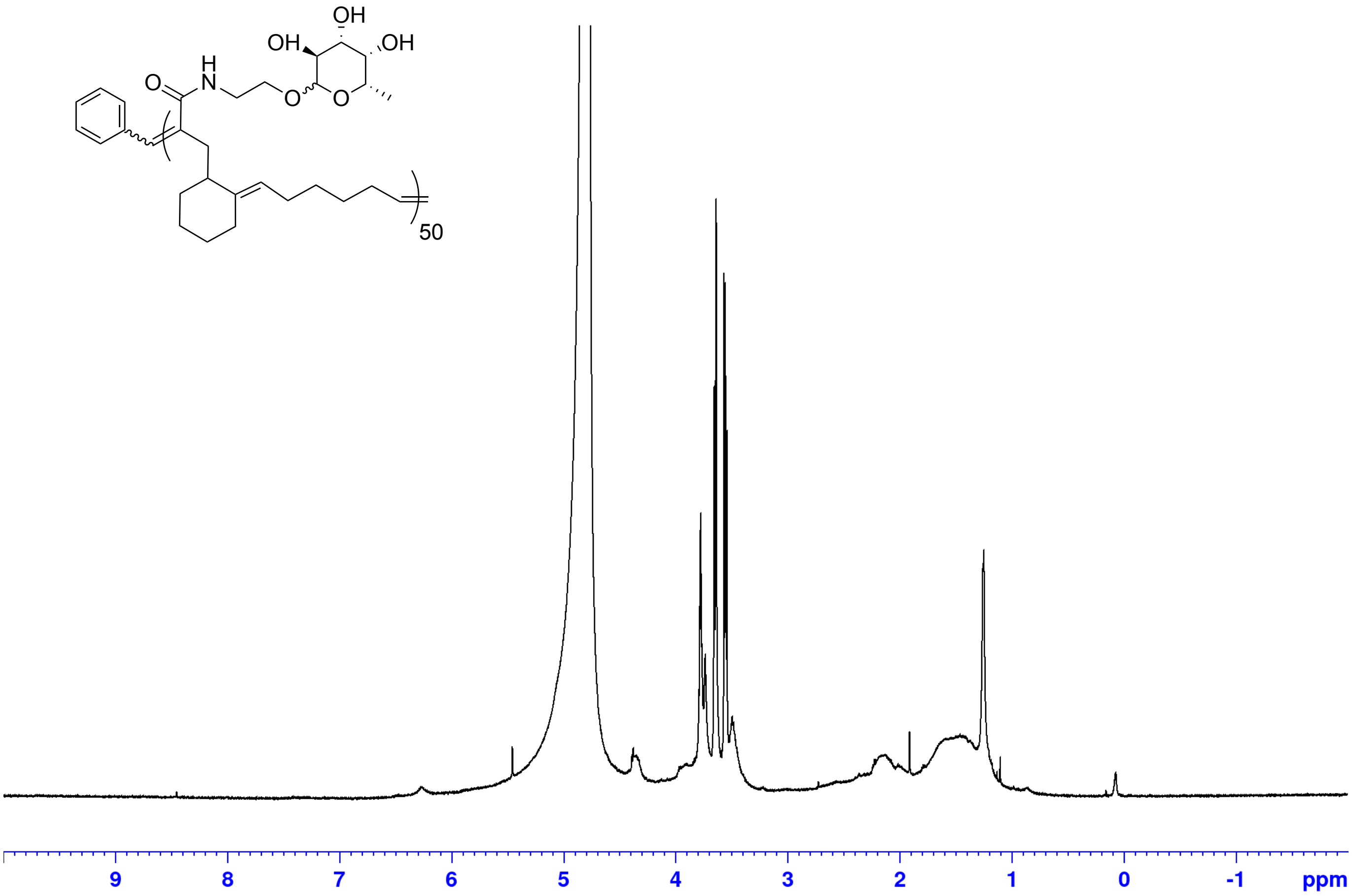

Figure S22. ^{1}H NMR (700 MHz, D_2O) spectrum of poly(**2b**)$_{50}$

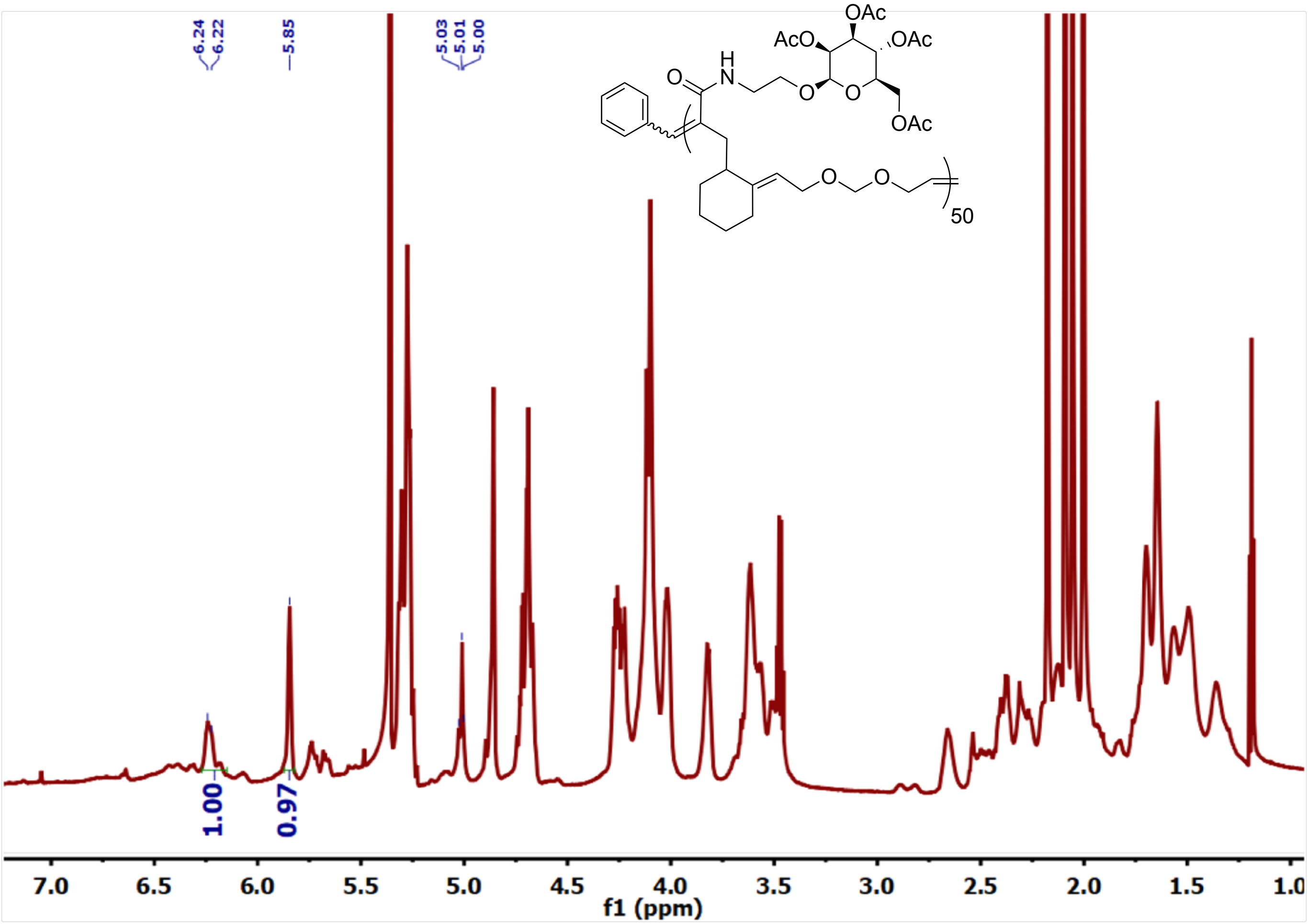

Figure S23. ^{1}H NMR (700 MHz, CD_2Cl_2) spectrum of poly(**3a′**)$_{50}$

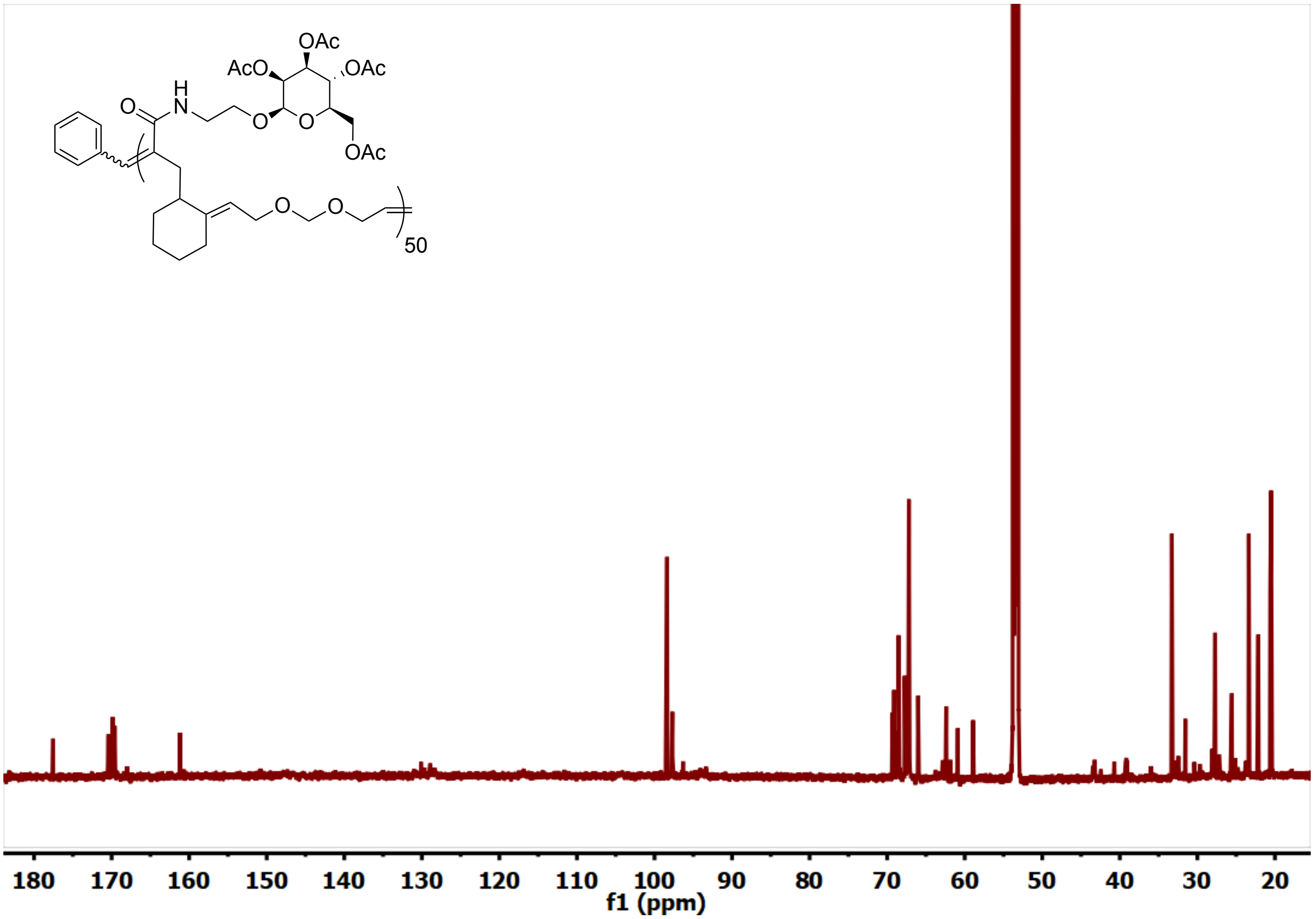

Figure S24. ^{13}C NMR (176 MHz, CD_2Cl_2) spectrum of poly(**3a′**)$_{50}$

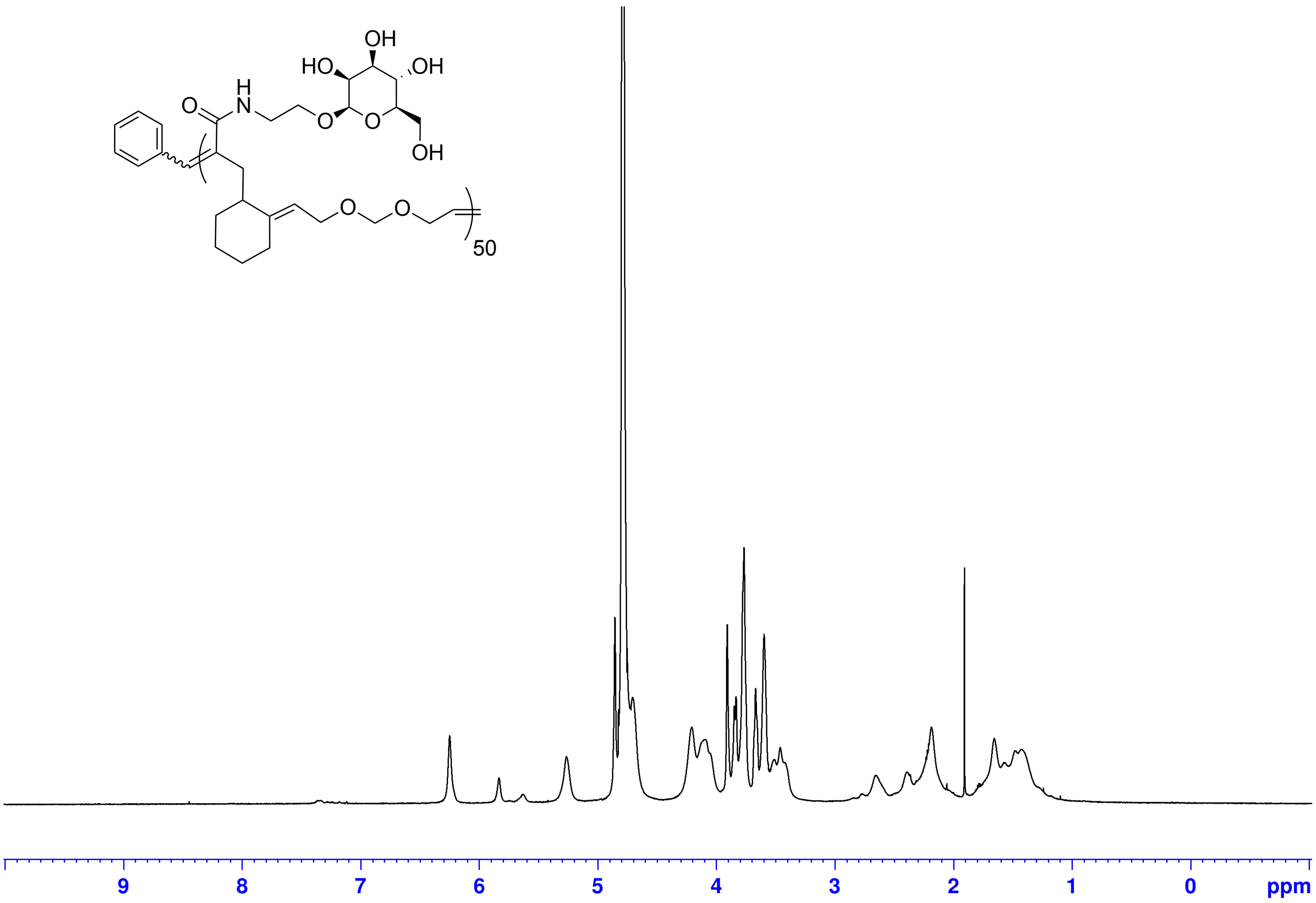

Figure S25. ^{1}H NMR (700 MHz, D_2O) spectrum of poly(**3a**)$_{50}$

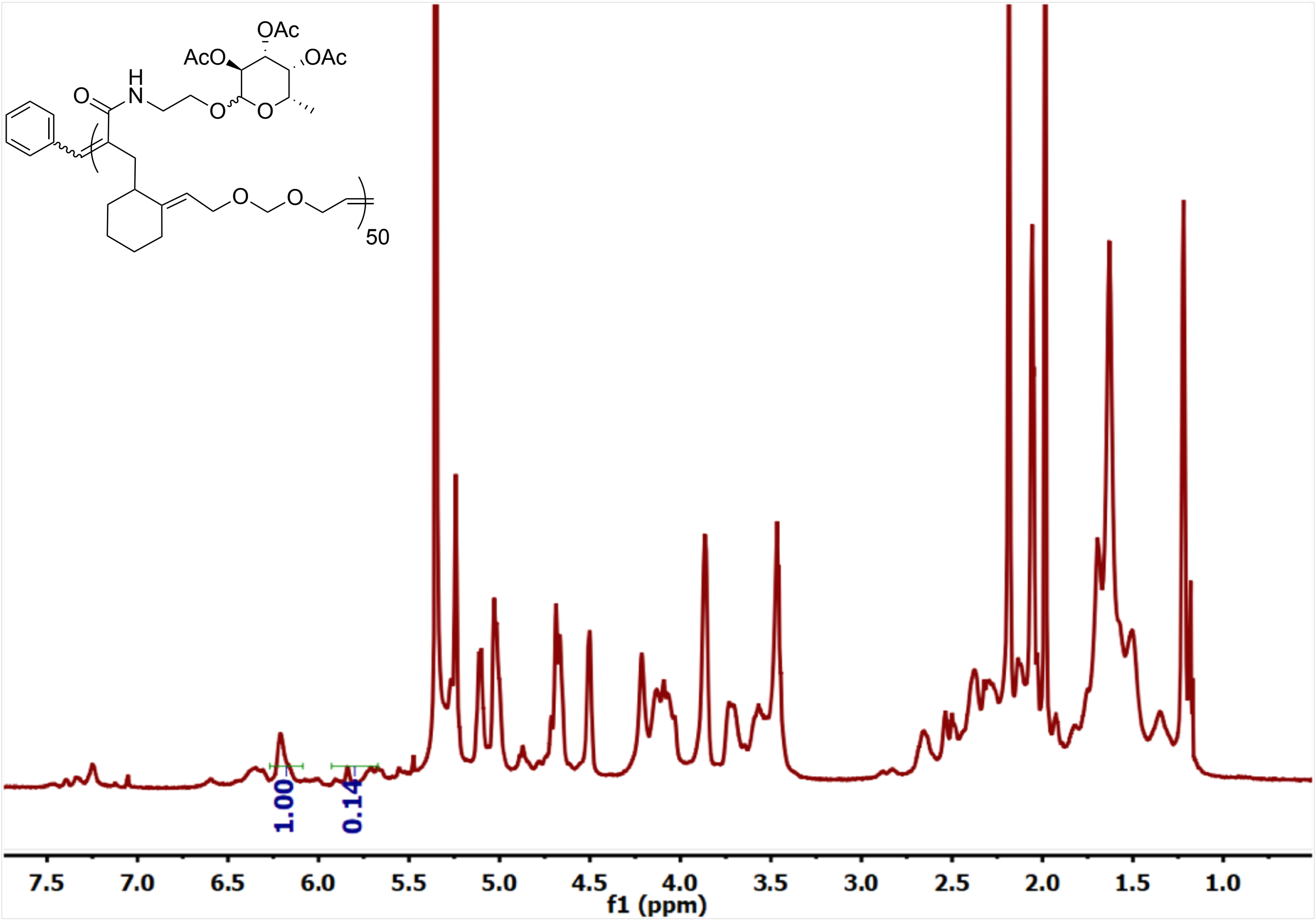

Figure S26. ^{1}H NMR (700 MHz, CD_2Cl_2) spectrum of poly(**3b′**)$_{50}$

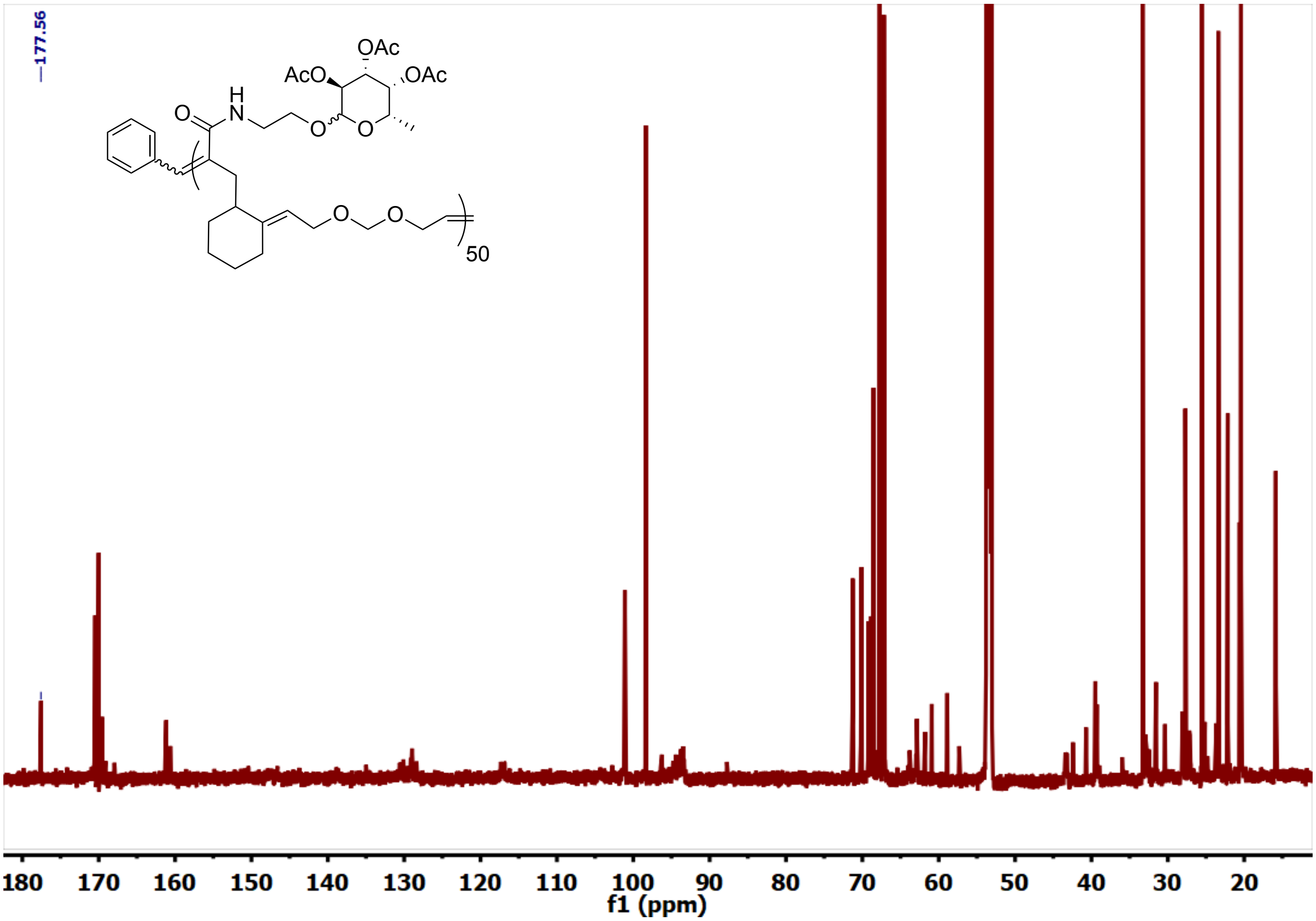

Figure S27. ^{13}C NMR (176 MHz, CD_2Cl_2) spectrum of poly(**3b′**)$_{50}$

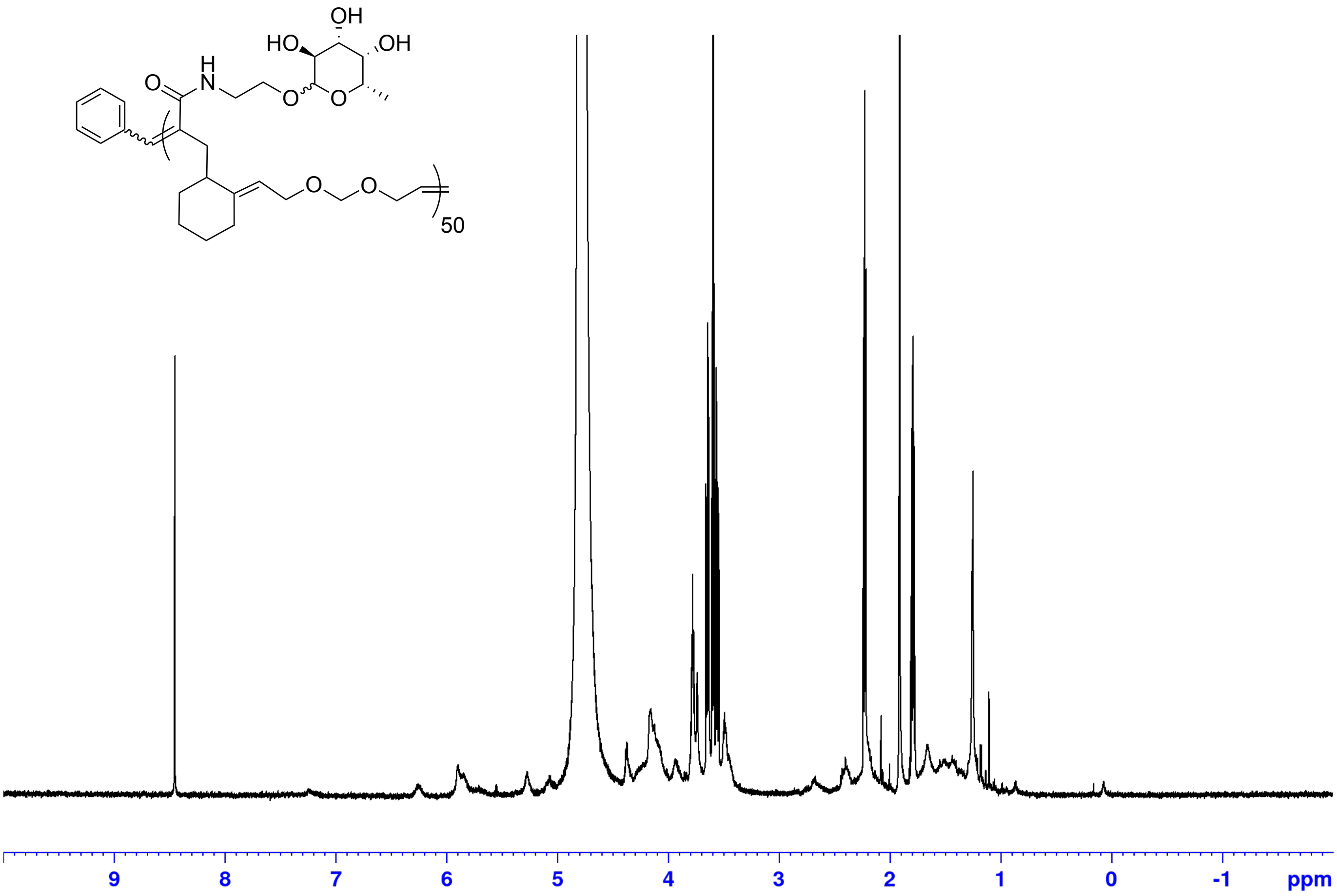

Figure S28. ^{1}H NMR (700 MHz, D_2O) spectrum of poly(**3b**)$_{50}$

References

1. Thermo Fisher Scientific Inc., *Protein Labeling Kits (For Alexa Fluor™, Pacific Blue™, Fluorescein-EX, and Oregon Green™ 488) User Guide MAN0019835*, Thermo Fisher Scientific. https://www.thermofisher.com/document-connect/document-connect.html?url=https://assets.thermofisher.com/TFS-Assets%2FLSG%2Fmanuals%2FMAN0019835_AlexaFluor_ProteinLabelingKits_UG.pdf (accessed February 2020).
2. Love, J. A.; Morgan, J. P.; Trnka, T. M.; Grubbs, R. H., A Practical and Highly Active Ruthenium-Based Catalyst that Effects the Cross Metathesis of Acrylonitrile. *Angew. Chem. Int. Ed.* **2002,** *41* (21), 4035-4037.
3. Wu, L.; Sampson, N. S., Fucose, Mannose, and β-N-Acetylglucosamine Glycopolymers Initiate the Mouse Sperm Acrosome Reaction through Convergent Signaling Pathways. *ACS Chem. Biol.* **2014,** *9* (2), 468-475.
4. Cervin, J.; Boucher, A.; Youn, G.; Björklund, P.; Wallenius, V.; Mottram, L.; Sampson, N. S.; Yrlid, U., Fucose-Galactose Polymers Inhibit Cholera Toxin Binding to Fucosylated Structures and Galactose-Dependent Intoxication of Human Enteroids. *ACS Infect. Dis.* **2020,** *6* (5), 1192-1203.
5. Hammouda, B., A New Guinier-Porod Model. *J. Appl. Cryst.* **2010,** *43*, 716-719.
6. Kikhney, A. G.; Svergun, D. I., A Practical Guide to Small Angle X-Ray Scattering (SAXS) of Flexible and Intrinsically Disordered Proteins. *FEBS Lett.* **2015,** *589* (19 Pt A), 2570-7.
